# Interplay between Public Attention and Public Emotion toward Multiple Social Issues on Twitter

**DOI:** 10.1371/journal.pone.0167896

**Published:** 2017-01-12

**Authors:** Tai-Quan Peng, Guodao Sun, Yingcai Wu

**Affiliations:** 1 Department of Communication, Michigan State University, East Lansing, Michigan, United States; 2 College of Information Engineering, Zhejiang University of Technology, Hangzhou, Zhejiang, China; 3 College of Computer Science, Zhejiang University, Hangzhou, Zhejiang, China; Universidad Veracruzana, MEXICO

## Abstract

This study aims to elucidate the intricate interplay between public attention and public emotion toward multiple social issues. A theoretical framework is developed based on three perspectives including endogenous affect hypothesis, affect transfer hypothesis, and affective intelligence theory. Large-scale longitudinal data with 265 million tweets on five social issues are analyzed using a time series analytical approach. Public attention on social issues can influence public emotion on the issue *per se*. Social issues interact with one another to attract public attention in both cooperative and competitive ways. Instead of a direct transfer from public emotion to public attention, the public emotion toward a social issue moderates the interaction between the issue and other issue(s).

## Introduction

The dynamic recruitment and distraction of public attention toward social issues has been an intriguing yet unanswered question in political communication research. Many scholars have developed conceptual and mathematical models to explicate the mechanisms that underlie the dynamics of public attention, such as issue-attention cycle [1, 2], threshold of public attention [3], and zero-sum game theory of public attention [4]. However, few studies have investigated the intricate link between what issues the general public thinks about (i.e., public attention) and how they feel about these issues (i.e., public emotion).

The missing link between public attention and public emotion has its conceptual and methodological causes. Conceptually, a century-long notion in political science claims that “wisdom is only possible when the emotions are silenced and when reason does all the talking” [5]. Given that public emotion is considered a destructive factor that can interfere in their information processing and decision making [6, 7], the public is assumed to process political information or make political judgments in an aseptic way that is uncontaminated by their affect [8]. Therefore, the role of emotion in political thinking and behavior has been largely ignored or under-studied [9–11]. Methodologically, most studies on emotion and politics employed retrospective self-reported measures to observe public emotion toward political objects, such as political campaigns [12], candidates [13], and social issues [14]. These self-reported measures are vulnerable to memory bias and experimenter demand effects and have coarse temporal granularity [15], which impedes the tracking of upstream and downstream emotional processes [10] and addressing of the interplay among emotion, political thinking, and behavior [9].

Psychological research has shown that emotion and cognition are interdependent with, rather than independent from, each other [5, 16, 17]. In particular, psychologists have investigated how the emotion individuals experience influences their allocation of attentional resources [18, 19]. Echoing these developments in psychological research, many researchers have begun to investigate the role of emotion in various political behaviors [9, 10] over the recent decades. Political scientists have proven emotion as an indispensable component in political judgment and behavior, such as susceptibility to political misinformation [20], candidate evaluation [21], presidential approval [22], political participation [23], and vote choice [24]. Moreover, the rapid development of machine learning and affective computing techniques empowers researchers to observe users’ attention and emotional states in an unobtrusive and direct way by mining large-scale, time-stamped textual information from social media [25].

Given that public attention on social issues is a fundamental element of responsible democracy and serves as a premise for subsequent political thinking and behavior, understanding how the dynamics of public attention on social issues will interplay with the dynamics of public emotion toward social issues is of theoretical significance. This study employs a theory-driven approach to develop research hypotheses on the intricate interplay between public attention and public emotion toward multiple social issues, which will be tested using large-scale longitudinal data collected from Twitter over a 12-month period.

## Literature Review and Research Hypotheses

Given that individuals can hold different emotional reactions toward various social issues [8], this study employs an issue-specific, instead of a generic, approach to examine the interplay between public emotion and public attention on multiple social issues. The study will draw on three theoretical perspectives, namely, endogenous affect hypothesis, affect transfer hypothesis, and affective intelligence theory, to develop research hypotheses about the interplay between public attention and public emotion toward social issues.

The first two perspectives address how public emotion and public attention mutually influence each other within a specific issue. In detail, the endogenous affect hypothesis focuses on how the public attention on a social issue influences the public emotion toward the issue *per se*, whereas the affect transfer hypothesis focuses on how the public emotion influences the public attention on the issue *per se* [24, 26].

The endogenous affect hypothesis argues that the pre-existing cognition toward an object induces corresponding emotional reactions toward the same object. However, different conceptualizations of cognition challenges the empirical testing of this hypothesis [16]. LeDoux (16) argued that if the cognition broadly includes both sensory information processing and higher mental functions, emotional processing will become highly dependent on cognitive processing. However, if the cognition includes only higher mental functions, emotional processing will not necessarily depend on prior cognitive processing. This study considers public attention on social issues as a sensory information processing that occurs in the human brain because such attention implies that the public perceives the significance of those issues to their well-being [26]. Therefore, public attention on social issues can be a precursor of public emotion toward social issues. In other words, the preexisting public attention on an issue will induce changes in the public emotion toward the issue *per se*. Relevant studies have not directly focused on the effect of public attention on public emotion, but the earlier candidate perception of citizens has been proven to influence their subsequent emotion toward the candidate [24], which is also found to be a rationalization of their previous evaluation of the candidate [27]. Based on the endogenous affect hypothesis, we propose the following:

H1: Public attention on a social issue can induce emotional response on the issue per se.

Affect transfer hypothesis is based on the classical idea of approach and avoidance tendency in psychological research. Individuals’ emotion toward an object can lead to behavioral response tendencies toward that object [28]. Such behavioral response may include both attention and action behaviors [29]. Positive emotion facilitates approach behavior, whereas negative emotion leads to avoidance behavior [30–32]. Based on these psychological findings, Brader [26] developed the affect transfer hypothesis, which argued that the emotion toward political objects can be directly transferred to relevant political behavior, such as political candidate evaluation [24] and political information seeking [33, 34].

In this study, the affect transfer hypothesis implies that the public emotion toward an issue can directly influence the public attention on the issue *per se*. In particular, when the public feels pleased or aroused toward an issue, they will adopt an approach tendency by focusing more attention to the issue. By contrast, when the public feels displeased or disenchanted toward an issue, they will adopt an avoidance tendency by focusing less attention to that issue. Therefore, we hypothesize the following:

H2: The more positive or aroused emotion individuals have toward a social issue, the greater public attention they will spend on the issue per se.

In addition to the within-issue interplay between public emotion and public attention, social issues interact with one another to gain public attention [4, 35]. The most intuitive type of interaction between social issues is competition, which implies that the public attention on one issue is increased at the expense of that on other issue(s) [4]. The competitive interaction between social issues is assumed in classical agenda-setting theory [36] and is explicitly tested as a zero-sum theory of agenda-setting [4, 35]. Nevertheless, recent studies that collected large-scale data from social media [37–39] found that competition is not the sole type of interaction that exists among social issues. Social issues can work together to achieve a win–win scenario or a cooperative interaction. Although clear-cut conclusion has not been reached on whether competition or cooperation dominates the interaction among social issues, researchers widely acknowledge that the dynamics of public attention on one issue intertwines with those on other issues. Therefore, we hypothesize the following:

H3: Public attention on one issue is contingent on public attention on another issue.

Affective intelligence theory suggests that the interaction between social issues is moderated by the public emotion toward social issues [12, 40]. This theory focuses on how emotion and reason interact with each other to produce a thoughtful and attentive citizenry, with a particular emphasis on “the dynamics between feeling and thing through which busy individuals come to pay some attention to the hubbub of the political world that swirls around them” [40]. Affective intelligence theory argues that two emotional subsystems, namely, the disposition and surveillance systems, operate in the human brain. The disposition system provides active feedback on ongoing behavior and motivates individuals to depend on their habits when making political judgment, whereas the surveillance system prompts individuals to uncouple their reliance on habit, shift their attention to the new stimuli, and develop stronger motivation for learning.

In line with affective intelligence theory, the negative or aroused emotion toward an issue can motivate individuals to marshal cognitive resources, which leads to a reallocation of attentive resources among social issues. When the negative emotion toward an issue alerts individuals to potential danger/problems that are embedded in such issue, they will stop their ongoing attention pattern and reallocate their attention among social issues by enhancing the interaction among these issues. Increased emotional arousal also motivates these individuals to expend their efforts and rapidly mobilize their cognitive resources to handle possible challenges in the issue [40, 41]. Therefore, we hypothesize the following:

H4: Negative or aroused emotion toward one issue will strengthen the competitive or cooperative interaction between the issue and other issue(s).

## Research Methods

This study focused on public emotion and public attention toward five social issues from May 2012 to April 2013. These five issues included Economy, Government-Politics (hereinafter labeled as “Politics”), Health, Job-Employment (hereinafter labeled as “Employment”), and Money-Spending (hereinafter labeled as “Spending”), which were found to be the top five most important problems facing the United States in public opinion polls [42]. The digital traces of users on Twitter were used to investigate how individuals would allocate their attention among the five issues and how they would feel about these issues.

### Data Collection and Cleaning

A keyword-based approach, which was used in empirical studies on Twitter [43, 44], was adopted to retrieve tweets. Following a bottom-up approach to generate search queries for social issues in [45], We derived an extensive list of 2,129 keywords on the five issues, which was reported in S1 Table.

Those tweets in English that contained the keywords in the list and were posted during the study period were retrieved from the Twitter Firehose. We obtained approximately 378 million tweets that were categorized into several issues according to the keywords included in the tweets. However, this keyword-based approach may include tweets that are irrelevant to the five issues under study. The support vector machine (SVM) classification method [46], a widely used automatic classification technique in machine learning, was then employed to clean up the retrieved tweets. Specifically, we built a training dataset by randomly selecting 1,000 tweets for each issue and manually coding each tweet as relevant or irrelevant for the associated issue. We constructed an SVM model for each issue by using 600 coded tweets and tested the model with the remaining 400 coded tweets. The SVM model requires that a tweet must be represented by a feature vector which was generated according to term frequency (occurrence of a word in a tweet). The average precision and recall rates were 0.80 and 0.84, respectively. We removed common words, such as stop words, before classification. The trained model was used to classify the tweets in each issue, which facilitated the removal of irrelevant tweets. A total of 265 million tweets were then retained for further analysis, of which 4% focused on Economy, 43% on Politics, 16% on Health, 25% on Employment, and 12% on Spending.

### Measurement and Aggregation of Public Attention and Public Emotion

Compared with solicited responses in public opinion polls, the self-initiated behavior of Twitter users (e.g., posting or retweeting a tweet on an issue) could better represent public attention in an unobtrusive way and with a fine-grained time scale [25]. In the study, we measured public attention on a social issue as a normalized score which refers to the percentage of the number of tweets related to a particular issue over the total number of tweets that were related to all five issues within a certain time window. The measurement of public attention as a normalized score has been a common practice in public opinion research [3, 35, 47]. A very important assumption underlying this normalization practice is that individuals in a society can have limited time and attention to process information they encounter. When individuals are faced with multiple social issues, they can only selectively pay attention to limited number of issues. This normalization practice can allow researchers to capture individuals’ selectivity in attention allocation.

We adopted a circumplex model of emotion [48] that describes emotion as a concept that comprised two dimensions, namely, arousal and valence. It has been found in psychological research that most of the variance in descriptions of emotions can be explained by these two dimensions: (1) valence, varying from negative to positive, and (2) arousal, varying from low to high [49–52]. Moreover, individuals’ attention control was found to be influenced by their emotional valence and arousal in psychological experiments [53, 54]. This two-dimension measurement of emotion has demonstrated great power in many empirical studies [55]. Therefore, in the study we measured public emotion toward each social issue into two dimensions, namely, emotional arousal and emotional valence.

A corpora-based approach, which assumes that those individuals who use the same language have similar conceptions of different discrete emotions [56], was employed to compute the emotional arousal and valence from the collected tweets. The Affective Norms for English Words (ANEW) [57], which was widely employed to measure emotion in various formats of online written expression, such as blog posts [58, 59], tweets [60], and discussions in online communities [22, 61], was used as the corpora. This corpora contains a large number of English words, with each word occupying a two-dimension measure of emotion (i.e., arousal and valence scores), which scores range from 1 to 9. For example, the mean arousal and valence scores of the word *happy* were 6.49 and 8.21 with standard deviations of 1.82 and 2.77. Each tweet was initially split into a sequence of words, and each word was pre-processed (e.g., chopping the suffix of the word) for further analysis. These words were then searched in the ANEW corpora. If the word was found in the corpora, we extracted the mean and standard deviations of the word’s arousal and valence scores. If a tweet contained no or only one ANEW word, the arousal and valence scores of the tweet would not be computed (i.e., considered missing) because we assumed that the number of words (i.e., less than two) was insufficient to estimate the emotion of the tweet.

Afterward, the arousal and valence scores of a tweet were estimated from those of the words that were extracted from the same tweet. In the estimation, we considered both the mean and standard deviation of the arousal and valence scores of each word. We assumed that the arousal and valence scores of each word follow a normal distribution. Therefore, a probability density function was adopted to estimate the probability for the arousal and valence scores of the word to fall at the corresponding mean values. The emotional arousal and valence scores of each tweet were estimated using the following equations in which the mean arousal and valence scores of each word in a tweet were initially weighted by their corresponding probabilities and then summed up:

$$T_{a}=\frac{\sum_{i}W_{i}^{a}P_{i}^{a}}{\sum_{i}P_{i}^{a}}\;where\;P_{i}^{a}=\int_{W_{i}^{a}-d_{w}}^{W_{i}^{a}+d_{w}}N(W_{i}^{a},\sigma_{a}^{2})$$

$$T_{v}=\frac{\sum_{i}W_{i}^{v}P_{i}^{v}}{\sum_{i}P_{i}^{v}}\;where\;P_{i}^{v}=\int_{W_{i}^{v}-d_{w}}^{W_{i}^{v}+d_{w}}N(W_{i}^{v},\sigma_{v}^{2})$$

where *T*_*a*_ and *T*_*v*_ represent the estimated arousal and valence scores of a tweet, $W_{i}^{a}$ and $W_{i}^{v}$ denote the mean of the arousal and valence scores of a word *W*_*i*_ in the tweet, and $P_{i}^{a}$ and $P_{i}^{v}$ denote the probability for the arousal and valence scores of the word to fall at the mean (i.e., $W_{i}^{a}$ and $W_{i}^{v}$). $W_{i}^{a}$ and *σ*_*a*_ are the mean and standard deviation that forms the normal distribution of arousal score, and $W_{i}^{v}$ and *σ*_*v*_ are the mean and standard deviation that forms the normal distribution of valence score of a word in ANEW dictionary. We set the value of *d*_*w*_ to small value (0.01 in this paper) to ensure the probability of the word *W*_*i*_’s arousal and valence score will fall at the mean.

All tweets were timestamped with 1-second precision. A daily interval was adopted to aggregate the public attention and public emotion toward social issues, thereby allowing us to obtain a reasonable number of observations and retain an adequate variance of study variables. The public attention on an issue per day was computed as the percentage of tweets on an issue that were posted within a day over all tweets that were posted within the day. Therefore, a time series of public attention with 365 time points was generated for each issue. The emotional arousal on an issue per day was computed as the average emotional arousal of tweets on an issue that were posted within the day, whereas the emotional valence on an issue per day was computed as the average emotional valence of tweets on an issue that were posted within the day. Therefore, two time series of emotional arousal and valence with 365 time points were generated for each issue.

### Real-Event Indicators

The effect of real-world cues in empirical research of public opinion must be controlled [62]. This study included six dummy variables to represent important real-life events that occurred between May 2012 and April 2013. These six events included the 9/11 memorial on September 11, 2012, Hurricane Sandy on October 29, 2012, the USA presidential elections on November 6, 2012, the Sandy Hook Elementary School shooting on December 14, 2012, the passing of the fiscal cliff bill by the US Senate on January 1, 2013, and the Boston Marathon bombing on April 15, 2013.

### Analytical Models and Design

To test the proposed hypotheses, a difference equation system was developed to examine the factors that underlie the changes in the emotional arousal and valence on a social issue *i* from time *t-1* to time *t* (*ΔArousal*_*i*_ and *ΔValence*_*i*_) and the factors that underlie the changes in the public attention on a social issue from time *t-1* to time *t (ΔAttention*_*i*_). The endogenous affect hypothesis (H1) is tested using Eqs (1) and (2), whereas the affect transfer hypothesis (H2) and affective intelligence theory (H3 and H4) are tested in the Eq (3).

$$\Delta Arousal_{i}=\beta_{0}+\beta_{1}Attention_{t-1,i}+\sum_{j=1}^{k}\beta_{2j}Arousal_{t-1,j}+\sum_{j=1}^{k}\beta_{3j}Valence_{t-1,j}+\beta_{4}Valence_{t-1,i}+\sum_{p=1}^{q}\beta_{5p}Events_{p}+e_{i}$$ (1)

$$\Delta Valence_{i}=\beta_{0}+\beta_{1}Attention_{t-1,i}+\sum_{j=1}^{k}\beta_{2j}Arousal_{t-1,j}+\sum_{j=1}^{k}\beta_{3j}Valence_{t-1,j}+\beta_{4}Arousal_{t-1,i}+\sum_{p=1}^{q}\beta_{5p}Events_{p}+e_{i}$$ (2)

$$\begin{array}{l} \Delta Attention_{i}=\beta_{0}+\beta_{1}Arousal_{t-1,i}+\beta_{2}Valence_{t-1,i}+\sum_{j=1}^{k}\beta_{3j}Attention_{t-1,j}+\sum_{j=1}^{k}\beta_{4j}(Arousal_{t-1,j}*Attention_{t-1,j})\\ \;+\sum_{j=1}^{k}\beta_{5j}(Valence_{t-1,j}*Attention_{t-1,j})+\sum_{j=1}^{k}\beta_{6j}Arousal_{t-1,j}+\sum_{j=1}^{k}\beta_{7j}Valence_{t-1,j}+\sum_{p=1}^{q}\beta_{8p}Events_{p}+e_{i} \end{array}$$ (3)

(i≠j,k=4,q=6)

In Eqs (1) and (2), *β*_*1*_ captures the effect of public attention on an issue at time *t-1* (*Attention*_*t-1*,*i*_) on the changes in the emotional arousal (*ΔArousal*_*i*_) and emotional valence (*ΔValence*_*i*_) toward the issue. The other terms in Eqs (1) and (2) control the effects of other possible confounding factors. Specifically, *β*_*2j*_ and *β*_*3j*_ in Eqs (1) and (2) control the effects of emotional arousal on other issues at time *t-1*(*Arousal*_*t-1*,*j*_) and those of emotional valence on other issues at time *t-1* (*Valence*_*t-1*,*j*_), respectively. In Eq (1), *β*_*4*_ controls the effect of emotional valence on the issue at time *t-1* (*Valence*_*t-1*,*i*_), whereas in Eq (2), *β*_*4*_ controls the effect of emotional arousal on the issue at time *t-1* (*Arousal*_*t-1*,*i*_). *β*_*5p*_ controls the effects of real-world indicators (*Events*_*p*_) in Eqs (1) and (2).

In Eq (3), the affect transfer hypothesis (H2) is tested using *β*_*1*_ and *β*_*2*_, which capture the main effects of emotional arousal on the issue at time *t-1* (*Arousal*_*t-1*,*i*_) and the emotional valence on the issue at time *t-1* (*Valence*_*t-1*,*i*_) on the change in public attention toward an issue *i* (*ΔAttention*_*i*_). *β*_*3j*_ captures the main effect of public attention on other issues at time *t-1* (*Attention*_*t-1*,*j*_) which is used to test empirically how social issues interact with one another to attract public attention (H3).

Affective intelligence theory is tested by including two interaction terms, namely, *β*_*4j*_ and *β*_*5j*_, in the Eq (3). *β*_*4j*_ captures the interaction effect between the emotional arousal on other issues and the public attention on other issues at time *t-1* (*Arousal*_*t-1*,j_*Attention_*t-1*,*j*_), and *β*_*5j*_ captures the interaction effect between the emotional valence on other issues and the public attention on other issues at time *t-1* (*Valence*_*t-1*,j_*Attention_*t-1*,*j*_). *β*_*6j*_ and *β*_*7j*_ control the effects of two moderators (i.e., *Arousal*_*t-1*,*j*_ and *Valence*_*t-1*,*j*_), and *β*_*8j*_ controls the effects of real-event indicators (*Events*_*p*_).

The three models comprise a simultaneous equation system that includes 15 equations for the changes in emotional arousal, emotional valence, and attention on five issues. The residual terms (*e*_*i*_) of these equations in the equation system tend to be correlated because those unconsidered factors that influence the residual term in one equation may influence the residual terms in the other equations [63]. Therefore, it is desirable to simultaneously estimate these equations in a system. We used the systemfit package in R [63], which allows us to specify multiple equations simultaneously and fit them with different estimation methods. Seemingly unrelated regression (SUR), also known as generalized least square estimation, was employed in this study because this method considers the covariance residual structure and produces efficient estimates [64].

## Analytical Findings

The findings section proceeds as follows. First, we describe the trends of public attention and public emotion toward the five social issues under study. Secondly, the overall model fit of the equation system is assessed. Then, we report our findings on the antecedents that underlie the changes in public emotion and public attention toward five issues. Finally, we would report how the real-event indicators would influence public emotion and public attention.

### Descriptive Findings

**Fig 1:**
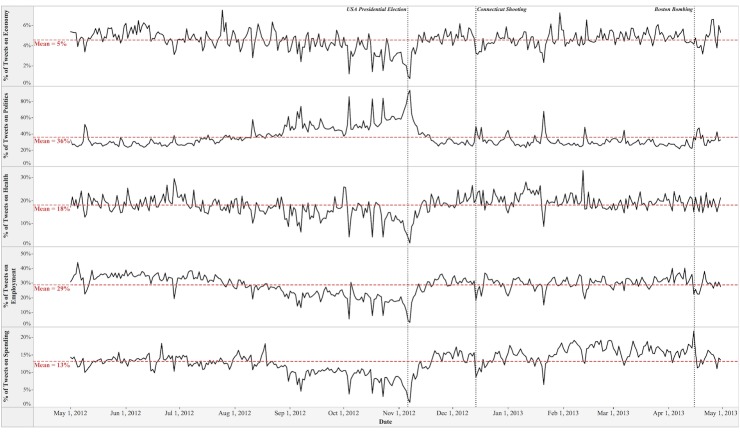
Dynamics of Public Attention toward Social Issues.

**Fig 2:**
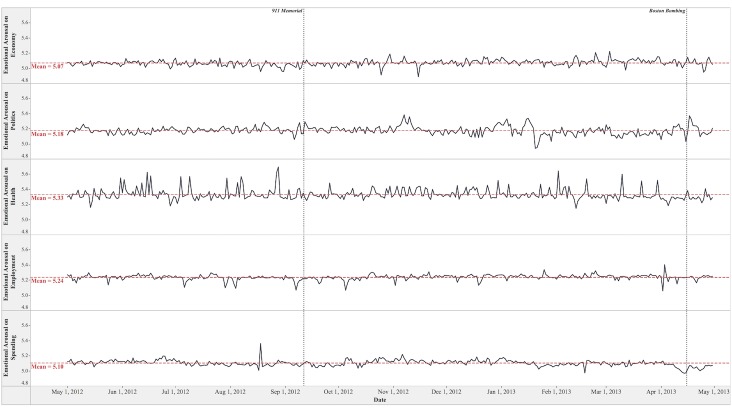
Dynamics of Emotional Arousal toward Social Issues.

**Fig 3:**
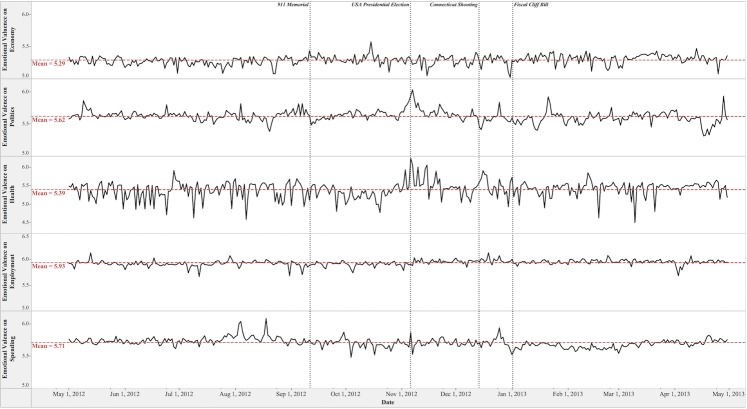
Dynamics of Emotional Valence toward Social Issues.

The trends of public attention and public emotion toward the five social issues are plotted in Figs 1–3, respectively. As shown in Fig 1, on average the Politics issue attracted the greatest share of public attention (36%) from May 2012 to April 2013, followed by the Employment issue (29%), the Health issue (18%), the Spending issue (13%), and the Economy issue (5%). The peak value of public attention on the Politics issue is 93%, which occurred on November 7^th^ 2012 when Mitt Romney conceded the election to Barack Obama. The peak values of public attention on other four issues are 44% for the Employment issue, 33% for the Health issue, 22% for the Spending issue, and 7% for the Economy issue.

On average, Twitter users possessed aroused and positive emotion toward the five social issues in the study period. The mean scores of emotional arousal on a 9-point scale are 5.33 (*S*.*D*. = 0.07) on the Health issue, 5.24 (*S*.*D*. = 0.04) on the Employment issue, 5.18 (*S*.*D*. = 0.05) on the Politics issue, 5.10 (*S*.*D*. = 0.04) on the Spending issue, and 5.07 (*S*.*D*. = 0.04) on the Economy issue. The mean scores of emotional valence on a 9-point scale are 5.93 (*S*.*D*. = 0.06) on the Employment issue, 5.71 (*S*.*D*. = 0.07) on the Spending issue, 5.62 (*S*.*D*. = 0.09) on the Politics issue, 5.39 (*S*.*D*. = 0.23) on the Health issue, and 5.29 (*S*.*D*. = 0.07) on the Economy issue.

### Bivariate Granger-Causality Tests

**Fig 4:**
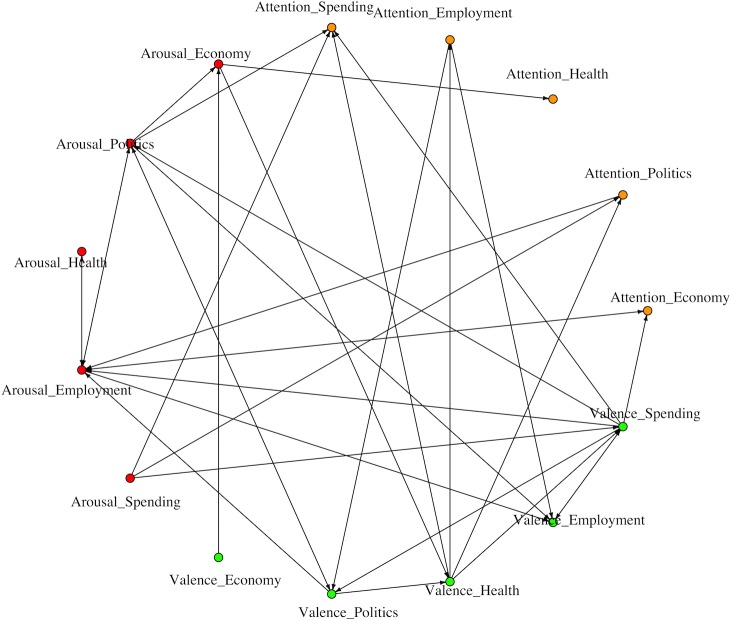
Statistically Significant Bivariate Granger-Causal Relationships.

Before SUR estimation is implemented to test the proposed hypotheses in the study, a bivariate Granger-causality test [65] is performed to examine the bivariate relationship among the fifteen time series. The results of bivariate Granger-causality test are summarized in S2 Table. Out of 210 Granger-causality tests among the 15 time series, 33 Granger-causality relationships are statistically significant which are summarized in Fig 4.

It is found that public attention on the Economy issue Granger-causes public attention on the Employment issue. Public attention on the Politics issue Granger-causes public arousal on the Employment issue. Public attention on the Employment issue Granger-causes public valence on the Politics and Employment issue.

Public arousal on the Economy issue Granger-causes public attention and valence on the Health issue. Public arousal on the Politics issue Granger-causes public attention on the Spending issue, public arousal on the Economy and Employment issue, and public valence on the Politics and Employment issue. Public arousal on the Health issue Granger-causes public arousal on the Employment issue. Public arousal on the Employment issue Granger-causes public attention on the Economy issue, public arousal on the Politics and Health issue, and public valence on the Employment issue. Public arousal on the Spending issue Granger-causes public attention on the Politics and Spending issue and public valence on the Spending issue.

Public valence on the Economy issue Granger-causes public arousal on the Economy issue. Public valence on the Politics issue Granger-causes public arousal on the Politics and Employment issues and public valence on the Health issue. Public valence on the Health issue Granger-causes public attention on the Politics, Employment, and Spending issues as well as public valence on the Spending issue. Public valence on the Employment issue Granger-causes public attention on the Spending issue. Public valence on the Spending issue Granger-causes public attention on the Economy and Spending issues, public arousal on the Politics and Employment issues, and public attention on the Politics issue.

### SUR Model Fit Assessment

SUR estimation results of the equation system are summarized in Tables 1–3, respectively. Before we report the hypothesis testing results, we should assess the overall model fit of the equation system with three measures, namely, the goodness-of-fit of the SUR equation system and the goodness-of-fit of specific equations in the system, the heteroscedasticity of residuals in specific regression equations, and the presence of autocorrelation among residuals in specific regression equation.

**Table 1:** SUR Estimation Results on the Change of Emotional Arousal on Social Issues.

Coefficients	Dependent Variables				
	ΔArousal_economy_	ΔArousal_politics_	ΔArousal_health_	ΔArousal_employment_	ΔArousal_spending_
**Intercept (*β***_***0***_**)**	–0.001(0.002)	0.001(0.003)	–0.001(0.005)	–0.0001(0.002)	–0.0003(0.002)
**Attention (*β***_***1***_**)**					
Economy_t-1_	0.332(0.290)	—-	—-	—-	—-
Politics_t-1_	—-	–0.006(0.027)	—-	—-	—-
Health_t-1_	—-	—-	–0.286(0.139)*	—-	—-
Employment_t-1_	—-	—-	—-	0.074(0.040)^†^	—-
Spending_t-1_	—-	—-	—-	—-	–0.059(0.078)
**Emotional Arousal (*β***_***2j***_**)**					
Economy_t-1_	—-	0.096(0.073)	0.094(0.127)	–0.023(0.058)	0.009(0.054)
Politics_t-1_	–0.054(0.046)	—-	–0.167(0.091)^†^	0.076(0.043)^†^	–0.048(0.040)
Health_t-1_	0.031(0.037)	0.022(0.043)	—-	0.018(0.034)	0.013(0.032)
Employment_t-1_	–0.032(0.073)	–0.100(0.085)	–0.128(0.149)	—-	0.050(0.062)
Spending_t-1_	0.026(0.066)	–0.032(0.075)	0.004(0.131)	–0.001(0.060)	—-
**Emotional Valence (*β***_***3j***_ **& *β***_***4***_**)**					
Economy_t-1_	–0.061(0.035)^†^	0.002(0.041)	–0.037(0.071)	0.009(0.033)	0.003(0.030)
Politics_t-1_	–0.017(0.029)	–0.009(0.034)	–0.067(0.060)	0.024(0.027)	–0.026(0.026)
Health_t-1_	0.006(0.012)	0.005(0.014)	0.09(0.021)***	–0.011(0.011)	–0.003(0.010)
Employment_t-1_	–0.018(0.047)	–0.076(0.056)	–0.084(0.096)	–0.075(0.041)^†^	–0.032(0.042)
Spending_t-1_	0.017(0.036)	0.024(0.042)	0.037(0.072)	0.016(0.034)	–0.022(0.030)
**Events (*β***_***5p***_**)**					
9/11 Memorial (September 11, 2012)	0.086(0.046)^†^	–0.005(0.053)	0.045(0.092)	0.024(0.042)	0.012(0.039)
Hurricane Sandy (October 29, 2012)	0.067(0.046)	0.037(0.053)	–0.087(0.092)	–0.034(0.042)	–0.006(0.039)
USA Presidential Elections (November 6, 2012)	0.015(0.046)	0.062(0.054)	0.121(0.093)	–0.002(0.042)	0.068(0.039)^†^
Sandy Hook Elementary School Shooting (December 14, 2012)	–0.062(0.045)	–0.071(0.053)	0.075(0.091)	0.009(0.042)	–0.009(0.039)
Passing of the Fiscal Cliff Bill by the Senate (January 1, 2013)	0.041(0.047)	–0.022(0.054)	–0.014(0.094)	0.009(0.043)	–0.004(0.040)
Boston Marathon Bombing (April 15, 2013)	0.044(0.046)	–0.144(0.053)**	0.067(0.093)	0.003(0.042)	0.008(0.039)
**Model Fit**					
Number of Cases	364	364	364	364	364
Durbin-Watson *d*	2.61	2.52	2.67	2.76	2.91
Adjusted *R*^2^	2.3%***	0.3%***	9.8%***	0.05%***	2.6%***

Note: Numbers enclosed in parentheses are the standard errors of estimates.

Level of significance

^†^ *p* < 0.10

* *p* < 0.05

** *p* < 0.01

*** *p* < 0.01.

**Table 2:** SUR Estimation Results on the Change of Emotional Valence on Social Issues.

Coefficients	Dependent Variables				
	ΔValence_economy_	ΔValence_politics_	ΔValence_health_	ΔValence_employment_	ΔValence_spending_
**Intercept (*β***_***0***_**)**	–0.001(0.004)	0.0001(0.004)	–0.004(0.014)	0.0002(0.003)	–0.001(0.003)
**Attention (*β***_***1***_**)**					
Economy_t-1_	1.089(0.516)*	—-	—-	—-	—-
Politics_t-1_	—-	–0.063(0.037) ^†^	—-	—-	—-
Health_t-1_	—-	—-	1.706(0.390)***	—-	—-
Employment_t-1_	—-	—-	—-	–0.039(0.057)	—-
Spending_t-1_	—-	—-	—-	—-	0.016(0.116)
**Emotional Arousal (*β***_***2j***_ **& *β***_***4***_**)**					
Economy_t-1_	–0.130(0.114)	0.053(0.105)	0.356(0.368)	0.018(0.083)	0.041(0.080)
Politics_t-1_	0.038(0.082)	0.033(0.075)	0.076(0.263)	0.037(0.061)	–0.009(0.060)
Health_t-1_	0.072(0.067)	–0.029(0.062)	1.123(0.192)***	–0.019(0.049)	–0.050(0.048)
Employment_t-1_	–0.131(0.132)	0.039(0.122)	0.114(0.428)	–0.078(0.090)	0.017(0.092)
Spending_t-1_	–0.054(0.117)	–0.008(0.106)	–0.301(0.378)	0.059(0.086)	–0.111(0.082)
**Emotional Valence (*β***_***3j***_**)**					
Economy_t-1_	—-	–0.026(0.058)	0.132(0.204)	–0.029(0.047)	–0.045(0.045)
Politics_t-1_	0.046(0.053)	—-	–0.036(0.174)	–0.036(0.039)	–0.048(0.039)
Health_t-1_	0.006(0.021)	0.008(0.020)	—-	–0.010(0.015)	–0.011(0.015)
Employment_t-1_	0.126(0.085)	–0.072(0.080)	–0.527(0.276)	—-	–0.126(0.062)*
Spending_t-1_	–0.032(0.064)	0.034(0.060)	–0.083(0.208)	0.012(0.049)	—-
**Events (*β***_***5p***_**)**					
9/11 Memorial (September 11, 2012)	0.159(0.082)^†^	–0.020(0.076)	–0.234(0.264)	0.038(0.060)	0.013(0.058)
Hurricane Sandy (October 29, 2012)	0.029(0.082)	–0.035(0.076)	0.106(0.265)	–0.084(0.060)	–0.006(0.058)
USA Presidential Elections (November 6, 2012)	–0.022(0.083)	0.106(0.077)	0.999(0.269)***	0.010(0.061)	0.241(0.059)***
Sandy Hook Elementary School Shooting (December 14, 2012)	–0.112(0.081)	–0.153(0.075)*	0.102(0.263)	–0.012(0.060)	0.028(0.058)
Passing of the Fiscal Cliff Bill by the Senate (January 1, 2013)	0.243(0.083)**	0.055(0.077)	0.192(0.271)	0.023(0.062)	–0.076(0.059)
Boston Marathon Bombing (April 15, 2013)	–0.078(0.083)	–0.007(0.077)	–0.077(0.267)	–0.040(0.061)	0.016(0.059)
**Model Fit**					
Number of Cases	364	364	364	364	364
Durbin-Watson *d*	2.78	2.53	2.72	2.66	2.43
Adjusted *R*^2^	3.6%***	1.3%***	19.0%***	3%***	5.8%***

Notes: Numbers enclosed in parentheses are the standard errors of estimates.

Level of significance

^†^ *p* < 0.10

* *p* < 0.05

** *p* < 0.01

*** *p* < 0.001.

**Table 3:** SUR Estimation Results on the Change of Public Attention on Social Issues.

Coefficients	Dependent Variables				
	ΔAttention_economy_	ΔAttention_politics_	ΔAttention_health_	ΔAttention_employment_	ΔAttention_spending_
**Intercept (*β***_***0***_**)**	0.000003(0.0004)	0.0001(0.003)	0.001(0.002)	–0.001(0.002)	–0.0004(0.001)
**Emotional Arousal (*β***_***1***_ **& *β***_***6j***_**)**					
Economy_t-1_	**–0.00002(0.010)**	*0*.*031(0*.*092)*	*–0*.*047(0*.*048)*	*–0*.*001(0*.*052)*	*0*.*012(0*.*023)*
Politics_t-1_	*0*.*007(0*.*008)*	**–0.036(0.068)**	*0*.*029(0*.*036)*	*–0*.*018(0*.*039)*	*0*.*026(0*.*017)*
Health_t-1_	*0*.*005(0*.*008)*	*0*.*029(0*.*060)*	**0.004(0.027)**	*–0*.*046(0*.*038)*	*0*.*004(0*.*016)*
Employment_t-1_	*0*.*026(0*.*013)*^*†*^	*–0*.*124(0*.*111)*	*0*.*117(0*.*062)*^*†*^	**–0.025(0.059)**	*0*.*009(0*.*030)*
Spending_t-1_	*–0*.*034(0*.011)^**^	*0*.*187(0*.*096)*^*†*^	*–0*.*028(0*.*051)*	*–0*.*068(0*.*055)*	–0.050(0.023)^*^
**Emotional Valence (*β***_***2***_ **& *β***_***7j***_**)**					
Economy_t-1_	–0.015(0.006)^**^	*0*.*100(0*.*054)*^*†*^	*0*.*001(0*.*029)*	*–0*.*089(0*.031)^**^	*0*.*003(0*.*014)*
Politics_t-1_	*0*.*001(0*.*005)*	**0.006(0.044)**	*–0*.*023(0*.*024)*	*0*.*008(0*.*026)*	*0*.*005(0*.*011)*
Health_t-1_	*–0*.*002(0*.*002)*	*–0*.*003(0*.*018)*	**–0.004(0.009)**	*0*.*007(0*.*010)*	*0*.*001(0*.*005)*
Employment_t-1_	*0*.*003(0*.*009)*	*–0*.*007(0*.*074)*	*0*.*010(0*.*042)*	**–0.045(0.040)**	*0*.*037(0*.*020)*^*†*^
Spending_t-1_	*–0*.*002(0*.*006)*	*0*.*049(0*.*053)*	*–0*.*013(0*.*028)*	*0*.*005(0*.*030)*	–0.036(0.014)^**^
**Attention (*β***_***3j***_**)**					
Economy_t-1_	——	2.511(0.525)^***^	**0.173(0.283)**	–0.771(0.317)^*^	**–0.054(0.160)**
Politics_t-1_	0.685(0.060)^***^	——	0.680(0.056)^***^	0.261(0.044)^***^	0.240(0.045)^***^
Health_t-1_	0.677(0.062)^***^	0.439(0.108)^***^	——	0.472(0.083)^***^^–^	0.275(0.058)^***^
Employment_t-1_	0.725(0.062)^***^	**-0.043(0.077)**	0.860(0.072)^***^	——	0.322(0.055)^***^
Spending_t-1_	0.711(0.070)^***^	**-0.266(0.175)**	0.845(0.111)^***^	0.577(0.122)^***^	——
Attention*Arousal Interaction (*β*_***4j***_**)**					
Economy_t-1_	——	7.414(7.420)	–4.044(4.477)	–0.802(4.908)	–2.459(1.926)
Politics_t-1_	0.072(0.043)^*†*^	——	–0.292(0.169)^†^	0.258(0.170)	–0.026(0.087)
Health_t-1_	–0.015(0.154)	–1.784(0.990)^*†*^	——	1.655(0.767)*	0.140(0.338)
Employment_t-1_	0.266(0.157)^*†*^	–1.010(0.958)	0.302(0.734)	——	0.436(0.348)
Spending_t-1_	0.347(0.222)	–2.430(1.745)	0.136(1.162)	1.945(1.249)	——
Attention*Valence Interaction (*β*_***5j***_**)**					
Economy_t-1_	——	1.839(4.191)	0.640(2.507)	–2.241(2.747)	–0.248(1.073)
Politics_t-1_	0.007(0.026)	——	–0.088(0.097)	0.061(0.099)	0.032(0.053)
Health_t-1_	0.013(0.039)	0.192(0.241)	——	–0.197(0.189)	–0.002(0.084)
Employment_t-1_	0.063(0.092)	–0.719(0.566)	0.540(0.436)	——	0.118(0.202)
Spending_t-1_	0.016(0.119)	–2.322(0.928)*	0.610(0.610)	1.615(0.665)*	——
**Events (*β***_***8p***_**)**					
9/11 Memorial (September 11,2012)	**0.009(0.007)**	**–0.042(0.064)**	**0.034(0.033)**	**–0.008(0.036)**	**0.006(0.016)**
Hurricane Sandy (October 29, 2012)	**–0.002(0.007)**	**–0.030(0.065)**	**–0.031(0.034)**	**–0.002(0.037)**	**0.005(0.017)**
USA Presidential Elections (November 6, 2012)	–0.018(0.008)^*^	0.220(0.066)^***^	**–0.056(0.035)**	–0.109(0.038)^**^	–0.038(0.017)^*^
Sandy Hook Elementary School Shooting (December 14, 2012)	–0.014(0.007)^*^	0.193(0.064)^**^	**0.010(0.033)**	–0.120(0.036)^***^	–0.068(0.016)^***^
Passing of the Fiscal Cliff Bill by the Senate (January 1, 2013)	**–0.002(0.007)**	**0.041(0.067)**	**–0.015(0.035)**	**–0.044(0.038)**	**0.021(0.017)**
Boston Marathon Bombing (April 15, 2013)	**–0.008(0.007)**	**0.158(0.065)**	**–0.064(0.034)**^*†*^	–0.129(0.037)^***^	0.043(0.016)^**^
**Model Fit**					
Number of Cases	364	364	364	364	364
Durbin-Watson *d*	2.18	2.27	2.08	2.21	2.29
Adjusted *R*^2^	28.9%^***^	20.2%^***^	28.5%^***^	22.5%^***^	20.7%^***^

Numbers enclosed in parentheses are the standard errors of coefficients.

The bolded numbers are the coefficients of the main-effect items, the italicized numbers are the coefficients of the moderators, and the underlined numbers are the coefficients of the interaction terms.

Level of significance

^†^ *p* < 0.10

* *p* < 0.05

** *p* < 0.01

*** *p* < 0.001.

First, the goodness-of-fit of the SUR equation system is evaluated with McElroy’s *R*^*2*^ [66], while the goodness-of-fit of specific equation is evaluated with adjusted *R*^*2*^ [67] which indicates the explanatory power of each equation in the equation system. The McElroy’s *R*^*2*^ of the SUR equation system in the study is 14%. All the fifteen equations in the SUR equation system are found to provide a significantly better fit to the observed data than the intercept-only model. However, the explanatory power of these fifteen equations varies. As shown in the lower panes of Tables 1 and 2, the ten equations, which account for the changes of emotional arousal and emotional valence toward the five issues, have quite small explanatory power. The adjusted *R*^*2*^ ranges from 0.05% to 9.8% in Table 1 and from 1.3% to 19% in Table 2. The five equations, which account for the change of public attention toward the five issues, have moderate explanatory power. The adjusted *R*^*2*^ ranges from 20.2% to 28.9% in Table 3.

**Fig 5:**
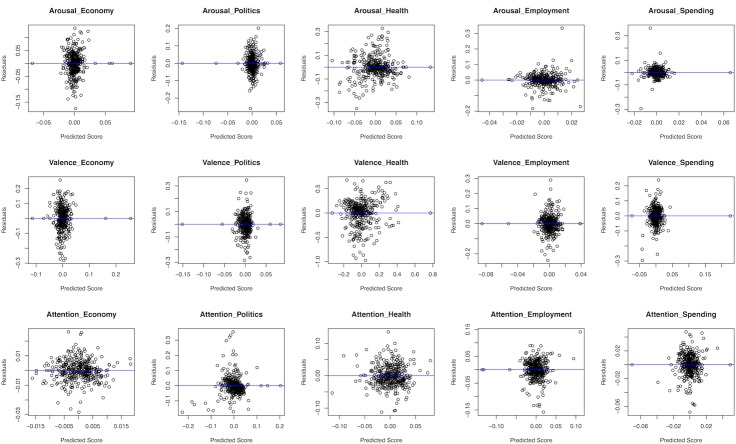
Residual Plots of SUR Estimations.

Secondly, the heteroscedasticity of residuals in each regression equation is assessed, which can help us determine if the coefficient estimates of each equation is biased or not [67]. If there is no heteroscedasticity among residuals of a regression equation, it suggests the coefficient estimates in the regression are unbiased. Otherwise, the coefficient estimates are biased. The heteroscedasticity of residuals can be assessed with residual plots in which the residuals of a regression equation are plotted against its predicted scores. If there are no clear patterns in residual plots, we can conclude that there is no heteroscedasticity among residuals of a regression equation and therefore the coefficient estimates in the regression are unbiased. In our study, we don’t identify any clear patterns from the residual plots of all fifteen equations as shown in Fig 5. In other words, the residuals of all fifteen equations are homoscedastic and the coefficient estimates in all equations are unbiased.

Third, the presence of autocorrelation among residuals is assessed with Durbin-Watson *d* (DW-*d*) statistics [68]. DW-*d* determines the presence of autocorrelation between adjacent residuals of the regression model, with *d* ranging from 0 (perfectly positive auto-correlation) to 4 (perfectly negative auto-correlation). As shown in the lower panes of Tables 1–3, the DW-*d* values of all fifteen equations range from 2.08 to 2.91, which is slightly above 2 (i.e., absence of auto-correlation). In other words, there are some negative autocorrelation remaining in the residuals of all fifteen regression equations, implying that the variance of the dependent variables in all equations (i.e., the change of public attention and public emotion toward five social issues) cannot be fully explained by the theoretical variables included in the model.

To summarize, the difference equation system in the study provided statistically significant model fit to the data and produced unbiased coefficient estimates, although some equations have quite limited explanatory power. Next, we would proceed to examine individual coefficients to test the proposed research hypotheses.

### Testing the Endogenous Affect Hypothesis

The direct effect of public attention on public emotion (H1), as suggested by the endogenous affect hypothesis, is partially supported in the study. As shown in Table 1, public attention on the Employment issue at an earlier period drives the public to feel more aroused toward the issue *per se* (*β* = 0.074, *p* < 0.10), whereas that on the Health issue causes the public to feel less aroused toward the issue *per se* (*β* = –0.286, *p* < 0.05). Public attention on the Economy, Politics, and Spending issues does not significantly cause the changes in emotional arousal toward the three issues.

Public attention on the Economy (*β* = 1.089, *p* < 0.05) and Health (*β* = 1.706, *p* < 0.001) issues causes the public to feel more positive toward the issues, as reported in Table 2. By contrast, public attention on the Politics issue causes the public to feel less positive toward the issue (*β* = –0.063, *p* < 0.10). Public attention on the Employment and Spending issues does not significantly cause the changes in their emotional valence toward the two issues.

### Testing the Affect Transfer Hypothesis and Affective Intelligence Theory

The affect transfer hypothesis, which argues that public emotion has a direct and positive effect on public attention toward social issues, is not supported. Instead, the public emotion toward an issue will moderate the interaction between the issue and other issues, thereby empirically supporting affective intelligence theory.

The emotional arousal and valence toward the Politics, Health, and Employment issues are independent from the changes in the public attention toward these issues. The emotional arousal toward the Economy issue does not significantly change the public attention on this issue. Interestingly, emotional valence and arousal negatively influence the changes in the public attention toward the Economy and Spending issues, which contradicts the affect transfer hypothesis. As reported in Table 3, the emotional valence toward the Economy issue decreases the attention on the issue *per se* (*β* = –0.015, *p* < 0.01), whereas the change in the public attention toward the Spending issue is negatively caused by the emotional arousal (*β* = –0.050, *p* < 0.05) and emotional valence (*β* = –0.036, *p* < 0.01) toward the Spending issue.

It is found that social issues can interact with one another in both competitive and cooperative ways to gain public attention. Competitive interaction, which refers that public attention on one issue at the cost of public attention on the other issue, is observed between Economy and Employment issues. Specifically, public attention toward the Economy issue drives a decreases of public attention toward the Employment issue (*β* = –0.771, *p* < 0.05), whereas that toward the Employment issue drives an increase of public attention toward the Economy issue (*β* = 0.725, *p* < 0.001).

Cooperative interaction, which refers that public attention on one issue will lead to an increase of public attention on the other issue, is found to be another type of interaction between multiple pairs of social issues in the study. Specifically, we found there are two subtypes of cooperative interaction: mutually cooperative interaction and one-way cooperative interaction. Mutually cooperative interaction, implying that the public attention toward two issues will contribute to each other, is observed among five pairs of issues, namely, Economy and Politics, Health and Politics, Health and Spending, Health and Employment, and Employment and Spending. One-way cooperative interaction, implying that the public attention toward one issue will contribute to that toward another issue but not vice versa, is observed among four pairs of issues, namely, Health and Economy, Politics and Employment, Politics and Spending, and Spending and Economy. Among these four pairs, the public attention toward the former issue increases the public attention toward the latter issue, while the public attention toward the latter issue does not significantly influence the public attention toward the former issue.

Consistent with affective intelligence theory, several interactions between social issues are moderated by the public emotion toward social issues. The emotional arousal toward the Politics issue strengthens the positive effect of public attention toward the Politics issue on the public attention toward the Economy issue (*β* = 0.072, *p* < 0.10). The emotional arousal toward the Health issue enhances the positive effects of public attention toward the Health issue on public attention toward the Employment issue (*β* = 1.655, *p* < 0.05). The emotional arousal toward the Employment issue strengthens the positive effect of public attention toward the Employment issue on public attention toward the Economy issue (*β* = 0.266, *p* < 0.10). The emotional valence toward the Spending issue negatively moderates the effect of public attention toward the Spending issue on public attention toward the Politics issue (*β* = –2.322, *p* < 0.05)

Significant moderation effects that contradict affective intelligence theory are also observed. The emotional arousal toward the Politics issue negatively moderates the positive effect of public attention toward the Politics issue on public attention toward the Health issue (*β* = –0.292, *p* < 0.10), whereas the emotional arousal toward the Health issue also negatively moderates the positive effect of public attention toward the Health issue on public attention toward the Politics issue (*β* = –1.784, *p* < 0.10). The emotional valence toward the Spending issue positively moderates the positive effect of public attention toward the Spending issue on public attention toward the Employment issue (*β* = 1.615, *p* < 0.05).

### Effects of Real-Event Indicators on Public Emotion and Public Attention

Among the six real-event indicators in this study, the 9/11 memorial in 2012 drives the public to feel more aroused and positive toward the Economy issue. Hurricane Sandy in 2012 does not significantly influence the public emotion toward all five social issues. The USA presidential elections in 2012 drives the public to feel more aroused toward the Spending issue and more positive toward the Health and Spending issues. The Sandy Hook Elementary School shooting in 2012 drives the public to feel less positive toward the Politics issue. The passing of the fiscal cliff bill in 2013 causes the public to feel more positive toward the Economy issue. The Boston Marathon bombing in 2013 drives the public to feel less aroused toward the Politics issue.

The 9/11 memorial, Hurricane Sandy, and the passing of the fiscal cliff bill do not significantly influence the public attention on all five social issues. The USA presidential election and the Sandy Hook Elementary School shooting increase the public attention on the Politics issue, but decrease the public attention on the Economy, Employment, and Spending issues. The Boston marathon bombing decreases the public attention on the Health and Employment issues, but increases the public attention on the Spending issue.

## Discussion and Conclusions

Public attention rarely focuses on a single issue for a long period, and the public tends to allocate their attention among several social issues in a dynamic way. However, the public was assumed to be a collection of cold-hearted individuals whose attention, perception, and behavior are insulated or hindered by their emotion [26]. Psychological literature has rejected such assumption and argued that the public is a collection of individuals who experience various emotional states toward several social issues [69]. However, we lack empirical evidence to verify the twists in the relationship between public emotion and public attention, namely, how the emotion individuals experience toward social issues influence their attention allocation and how the attention that individuals allocate to these issues will influence their emotion.

We fill the research gap by performing an integrated test of three perspectives that concern the role of emotion in politics. The large-scale longitudinal data from Twitter and the time series analytical approach allow us to investigate the subtle interplay between public attention and public emotion within an issue and the intricate role of public emotion in the allocation of attention among several social issues. Our findings have theoretical and methodological significance, which can advance our knowledge about the roles of public emotion in political communication as well as the driving mechanisms that underlie the dynamics of public attention.

The first two hypotheses concern the endogenous mutual influence between public emotion and public attention within an issue. Our findings partially support the endogenous affect hypothesis, which argues that the public attention on a social issue will change the public emotion toward the issue *per se*. By contrast, we reject the affect transfer hypothesis, which argues that the aroused and positive emotion toward an issue induces a positive change in the public attention on the issue *per se*. Moreover, the issue-specific approach and the two-dimension measure of public emotion allow us to delineate the boundary of these two hypotheses.

The attention-solicited change in public emotion is found to exist on four issues, namely, Economy, Employment, Health, and Politics, but such change is not detected on the Spending issue. Moreover, the direction of such change varies among several issues and between emotional dimensions. Individuals’ increasing attention on the Employment issue would make them feel more aroused toward the issue, while their increasing attention on the Health issue will make them feel less aroused toward the issue. Individuals’ increasing attention on the Economy and Health issues will make them feel more positive toward the issue, while individuals’ increasing attention on the Politics issue will make them feel less positive toward the issue. This finding confirms an argument in psychological research that the emotion of individuals is specific to an issue or event [70]. Such an issue-specific approach is necessary in future research to understand fully the intricate role of emotion in political communication.

Emotion is argued to “profoundly influence processes of engagement (interest), attention, information seeking, and memory—in short, what citizens are exposed to and what they learn” [10], but the affect transfer hypothesis is rejected in this study. When the effects of public attention toward other issues and potential confounding factors are controlled, the effects of public emotion on public attention are not significantly positive for all five issues. Consistent with the findings of previous studies [26, 71], this study casts doubt on the conventional wisdom that the positive or aroused emotion toward a social issue can be mechanically transferred to attract more attention toward a particular issue.

Nevertheless, our findings do not suggest that the public emotion toward social issues will not lead to the change of public attention. Interestingly, the emotional valence toward the Economy issue as well as the emotional arousal and valence toward the Spending issue induce negative changes in the public attention toward both issues. In other words, the positive emotion toward these two issues dilutes the public attention on these issues. Further studies must further explore the influential mechanism of public emotion on public attention within an issue.

Our findings confirm that the rise and fall of public attention toward a social issue is not an independent process and that social issues interact with one another to attract public prominence. The large-scale digital footprint on Twitter also helps us identify three types of interaction (e.g., competitive, mutually cooperative, and one-way cooperative) among the five social issues under study. Our findings differ from those of earlier studies that identify competition as the mainstream type of interaction among social issues [4, 35].

Such difference may be attributed to the research context of this study, which draws on user-generated content on Twitter to measure the public attention and public emotion toward social issues. Twitter users use closely related words or adopt similar linguistic styles to express their interest in related issues (e.g., Economy, Employment, and Spending), which makes it easier to capture the cooperation, instead of competition, between social issues [38, 39]. The contrasting findings may also be attributed to the pairwise interaction that is examined in this study. We only focused on the interaction between two social issues. However, it has been found that two related social issues can work jointly to attract public attention from the third issue [38]. In other words, higher-order and more complicated interaction between multiple social issues can exist among social issues to recruit public attention.

Therefore, we cannot conclude that cooperation, rather than competition, is the dominant type of interaction among social issues because we only consider the interaction among five specific issues within a specific time window. However, our findings imply that competition must not be the sole type of interaction among social issues, especially on social media where users are confronted with a preponderance of attentional choices and have a tight grip on their attentive issues. Future political communication research must give equal attention to cooperative and competitive types of interaction among social issues and consider the higher-order interactions among social issues.

The interaction among social issues is assumed to result solely from the rational choices of individuals. Nevertheless, we find that the public emotion toward social issues has a critical role in such interactions by contributing to the development of a realistic theory of political judgment [40]. Instead of promoting a direct transfer from public emotion to public attention, the public emotion toward an issue moderates the interaction between the issue and other issue(s). The emotion-moderated interaction among social issues provides mixed support to affective intelligence theory.

Consistent with affective intelligence theory, the aroused emotion toward an issue (i.e., Politics, Health, and Employment) and the negative emotion toward an issue (i.e., Spending) enhance the interaction between the issue and other issues. In particular, the aroused and negative feelings serve as signals that are activated by individuals to re-assess their information environment and adjust their pre-existing attention allocation strategy [40]. This kind of re-assessment and adjustment engages individuals to utilize cognitive resources to process relevant information, which further intensifies the interaction between social issues.

Inconsistent with affective intelligence theory, the aroused emotion toward the Politics and Health issues negatively moderates the positive mutual interaction between Politics and Health, whereas the positive emotion toward the Spending issue strengthens its interaction with the Employment issue. Accounting for these contradictory findings is beyond the scope of this study. Nevertheless, these findings cannot be considered a complete rejection of affective intelligence theory. Future studies must be conducted to find out for which issues and under what conditions affective intelligence theory can work, which is at the core of theory development in social sciences [67] and indicates the maturity and sophistication of a theory [72].

## Limitations and Future Research

The limitations of this study are as follows. The first limitation is the aggregate-level analysis that is adopted in the study. The aggregate data are subject to so-called “ecological fallacy” [73], as it makes two stronger assumptions about the uniformity of the population. The first assumption is that the causal relationship specified at the aggregate level should have a psychological mechanisms operating at the individual level, while the second assumption is that the strength and direction of the relationship found at the aggregate level should be comparable to that at the individual level. The first assumption is not a concern in the study, because the hypothesized relationships between public attention and public emotion are developed based on psychological theories at the individual level and are proved to be plausible at the individual level. Nevertheless, the second assumption that the aggregate-level findings derived from the current study can be generalized to the individual level should be empirically tested in the future. Meanwhile, we have to admit that the individual-level analysis of the dynamic interplay between public attention and public emotion poses very demanding requirements to researchers’ data collection, as we need to continuously observe a random sample of individuals for an adequately long time frame. How to draw a random sample of user from a population on social media and how to access streaming behavioral data of a specific group of users via public API provided by social media companies are both challenging tasks in social media studies [74, 75].

The second limitation is the small explanatory power of the two models which are proposed to account for the changes of emotional arousal and emotional valence toward the five social issues. Theoretically, this finding suggests that the endogenous affect perspective could not adequately explain the change of emotional arousal and valence toward social issues. Future studies should take into account other important variables that are relevant to the change of public emotion. Empirically, the proposed model in our study only consider the linear relationship between variables. Future studies could also explore the nonlinear relationships between public emotion and other relevant variables with theoretical backup.

The third limitation is the top-down approach in determining the social issues under study and the keyword-based approach adopted retrieve issue-related tweets from Twitter. Despite its efficiency, this approach underestimates, to an unknown extent, the size of the issue space unless an exhaustive list of keywords is used. Moreover, the relative share of each searched issue on the issue space tends to be biased to an unknown extent. Future research are needed to empirically examine the robustness of our findings when other social issues are taken into account.

Last but not least, although the two-dimension measurement of public emotion is well-grounded and widely adopted in empirical studies, this measurement prevents researchers from examining the effects of subtle emotional states, such as happiness, anger, anxiety, depression, and fatigue. Future research are needed to employ other operationalization of public emotion to investigate the relationship between public attention and specific emotional states. Moreover, as individuals in different cultures may have different attention allocation patterns and emotion expression patterns, future studies can examine if the findings of the study can be replicated in other cultural contexts or not.

## Supporting Information

S1 Table List of Keywords on Five Issues. (DOCX)

S2 Table Bivariate Granger-Causality Test Results. (DOCX)

## References

[1] Downs A. Up and down with ecology: the issue attention cycle. Public Interest. 1972;28:38–50.

[2] Henry GT, Gordon CS. Tracking issue attention: Specifying the dynamics of the public agenda. Public Opinion Quarterly. 2001;65(2):157–77. 11420754 10.1086/322198

[3] Neuman WR. The threshold of public attention. Public Opinion Quarterly. 1990;54(2):159–76.

[4] Zhu JH. Issue competition and attention distraction—a Zero-Sum Theory of Agenda-Setting. Journalism Quarterly. 1992;69(4):825–36.

[5] Gross JJ. The emerging field of emotion regulation: an integrative review. Review of general psychology. 1998;2(3):271–99.

[6] Marcus GE. Emotions in politics. Annual Review of Political Science. 2000;3(1):221–50.

[7] Marcus GE. The Psychology of emotion and politics In: Huddy L, Sears D, Jervis R, editors. Oxford Handbook of Political Psychology. New York: Oxford University Press; 2003 p. 182–221.

[8] Conover PJ, Feldman S. Emotional reactions to the economy: I'm mad as hell and I'm not going to take it anymore. American Journal of Political Science. 1986;30(1):50–78.

[9] Brader T, Marcus GE. Emotion and politcal psychology In: Huddy L, Sears DO, Levy JS, editors. The Oxford Handbook of Political Psychology. 2nd ed. New York: Oxford University Press; 2013 p. 165–204.

[10] Brader T, Marcus GE, Miller KL. Emotion and public opinion In: Edwards GC, Jacobs LR, Shapiro RY, editors. The Oxford Handbook of American Public Opinion and the Media. New York: Oxford Univeristy Press; 2011 p. 385–401.

[11] Neuman WR, Marcus GE, Crigler AN, Mackuen M. Theorizing affect's effects In: Neuman WR, Marcus GE, Crigler AN, Mackuen M, editors. The affect effect: Dynamics of emotion in political thinking and behavior. Chicago: University of Chicago Press; 2007 p. 1–20.

[12] Marcus GE, Mackuen MB. Anxiety, enthusiasm, and the vote: The emotional underpinnings of learning and involvement during presidential campaigns. The American Political Science Review. 1993;87(3):672–85.

[13] Redlawsk DP, Civettini AJW, Emmerson KM. The affective tipping point: Do motivated reasoners ever “get it”? Political Psychology. 2010;31(4):563–93.

[14] Gross K, Brewer PR, Aday S. Confidence in government and emotional responses to terrorism after September 11, 2001. American Politics Research. 2009;37(1):107–28.

[15] Golder SA, Macy MW. Diurnal and seasonal mood vary with work, sleep, and daylength across diverse cultures. Science. 2011;333(6051):1878–81. 10.1126/science.1202775 21960633

[16] LeDoux JE. Emotion: Clues from the brain. Annual Review of Psychology. 1995;46(1):209–35. 10.1146/annurev.ps.46.020195.001233 7872730

[17] Simon HA. Motivational and emotional controls of cognition. Psychological Review. 1967;74(1):29–39. 5341441 10.1037/h0024127

[18] Salovey P. Mood-induced self-focused attention. Journal of personality and social psychology. 1992;62(4):699–707. 1583593 10.1037//0022-3514.62.4.699

[19] Mor N, Winquist J. Self-focused attention and negative affect: A meta-analysis. Psychol Bull. 2002;128(4):638–62. 12081086 10.1037/0033-2909.128.4.638

[20] Weeks BE. Emotions, partisanship, and misperceptions: How anger and anxiety moderate the effect of partisan bias on susceptibility to political misinformation. Journal of Communication. 2015;65(4):699–719.

[21] Holbert RL, Hansen GJ, Caplan SE, Mortensen S. Presidential debate viewing and Michael Moore's Fahrenheit 9–11: A study of affect-as-transfer and passionate reasoning. Media Psychology. 2007;9(3):673–94.

[22] González-Bailón S, Banchs RE, Kaltenbrunner A. Emotions, public opinion, and U.S. presidential approval rates: A 5-year analysis of online political discussions. Human Communication Research. 2012;38(2):121–43.

[23] Jones PE, Hoffman LH, Young DG. Online emotional appeals and political participation: The effect of candidate affect on mass behavior. New Media & Society. 2012.

[24] Ladd JM, Lenz GS. Reassessing the role of anxiety in vote choice. Political Psychology. 2008;29(2):275–96.

[25] Golder SA, Macy MW. Digital footprints: Opportunities and challenges for online social research. Annual Review of Sociology. 2014;40(1):129–52.

[26] Brader T. Campaign for hearts and minds: How emotional appeals in political ads work. Chicago: University of Chicago Press; 2006.

[27] Rahn WM, Jon AK, Breuning M. Rationalization and derivation processes in survey studies of political candidate evaluation. American Journal of Political Science. 1994;38(3):582–600.

[28] Lazarus RS. Emotion and adaptation. Oxford, England: Oxford University Press; 1991.

[29] Fiedler K. Emotional mood, cognitive style, and behavior regulation In: Fiedler K, Forgas J, editors. Affect, cognition and social behavior: New evidence and integrative attempts. Toronto, Canada: C. J. Hogrefe; 1988 p. 100–19.

[30] Fredrickson BL. The role of positive emotions in positive psychology—The broaden-and-build theory of positive emotions. American Psychologist. 2001;56(3):218–26. 11315248 10.1037//0003-066x.56.3.218 PMC3122271

[31] Bradley MM. Natural selective attention: Orienting and emotion. Psychophysiology. 2009;46(1):1–11. 10.1111/j.1469-8986.2008.00702.x 18778317 PMC3645482

[32] Lang PJ. The emotion probe—Studies of motivation and attention. American Psychologist. 1995;50(5):372–85. 7762889 10.1037//0003-066x.50.5.372

[33] Brader T, Valentino NA, Suhay E. What triggers public opposition to immigration? Anxiety, group cues, and immigration threat. American Journal of Political Science. 2008;52(4):959–78.

[34] Valentino NA, Hutchings VL, Banks AJ, Davis AK. Is a worried citizen a good citizen? Emotions, political information seeking, and learning via the Internet. Political Psychology. 2008;29(2):247–73.

[35] McCombs M, Zhu JH. Capacity, diversity, and volatility of the public agenda—Trends from 1954 to 1994. Public Opinion Quarterly. 1995;59(4):495–525.

[36] McCombs ME, Shaw DL. The agenda-setting function of mass media. Public Opinion Quarterly. 1972;36(2):176–87.

[37] Coscia M, editor Competition and success in the meme pool: a case study on Quickmeme.com. AAAI Conference on Weblogs and Social Media; 2013; Boston, USA.

[38] Sun GD, Wu YC, Liu SX, Peng TQ, Zhu JJH, Liang RF. EvoRiver: Visual analysis of topic coopetition on social media. IEEE Transactions on Visualization and Computer Graphics. 2014;20(12):1753–62. 10.1109/TVCG.2014.2346919 26356889

[39] Myers S, Leskovec J, editors. Clash of the contagions: Cooperation and competition in informatno diffusion. IEEE International Conference on Data Mining; 2012; Brussels, Belgium.

[40] Marcus GE, Neuman WR, Mackuen M. Affective intelligence and political judgment. Chicago: The University of Chicago Press; 2000.

[41] Graber DA. The road to public surveillance: Breeching attention thresholds In: Neuman WR, Marcus GE, Crigler AN, Mackuen M, editors. The affect effect: Dynamics of emotion in political thinking and behavior. Chicago: The University of Chicago Press; 2007 p. 265–90.

[42] Most Important Problem [Internet]. 2016 [cited Nov 28, 2016]. Available from: http://www.gallup.com/poll/1675/most-important-problem.aspx.

[43] Neuman WR, Guggenheim L, Mo Jang S, Bae SY. The dynamics of public attention: Agenda-setting theory meets big data. Journal of Communication. 2014;64(2):193–214.

[44] Guggenheim L, Jang SM, Bae SY, Neuman WR. The dynamics of issue frame competition in traditional and social media. The ANNALS of the American Academy of Political and Social Science. 2015;659(1):207–24.

[45] Qin J, Peng T-Q. Googling environmental issues Web search queries as a measurement of public attention on environmental issues. Internet Research. 2016;26(1):57–73.

[46] Chang C-C, Lin C-J. LIBSVM: A library for support vector machines. ACM Transactions on Intelligent Systems and Technology. 2011;2(3):1–27.

[47] Kleinnijenhuis J, Schultz F, Oegema D. Frame complexity and the financial Crisis: A comparison of the United States, the United Kingdom, and Germany in the period 2007–2012. Journal of Communication. 2015;65(1):1–23.

[48] Russell JA. A circumplex model of affect. Journal of personality and social psychology. 1980;39(6):1161–78.

[49] Mehrabian A, Russell JA. An approach to environmental psychology. Cambridge, MA: MIT Press; 1974.

[50] Russell JA. Core affect and the psychological construction of emotion. Psychological review. 2003;110(1):145 12529060 10.1037/0033-295x.110.1.145

[51] Anders S, Lotze M, Erb M, Grodd W, Birbaumer N. Brain activity underlying emotional valence and arousal: A response‐related fMRI study. Human brain mapping. 2004;23(4):200–9. 10.1002/hbm.20048 15449355 PMC6871723

[52] Barrett LF. Are emotions natural kinds? Perspectives on psychological science. 2006;1(1):28–58. 10.1111/j.1745-6916.2006.00003.x 26151184

[53] Derryberry D, Tucker DM. Motivating the focus of attention In: Niedenthal PM, Kitayama S, editors. The heart's eye: Emotional influences in perception and attention. San Diego, CA: Academic Press; 1994 p. 167–96.

[54] Jefferies LN, Smilek D, Eich E, Enns JT. Emotional valence and arousal interact in attentional control. Psychological Science. 2008;19(3):290–5. 10.1111/j.1467-9280.2008.02082.x 18315803

[55] Stevenson R, Mikels J, James T. Characterization of the Affective Norms for English Words by discrete emotional categories. Behav Res. 2007;39(4):1020–4. 10.3758/bf03192999 18183921

[56] Calvo RA D 'Mello S. Affect detection: An interdisciplinary review of models, methods, and their applications. IEEE Transactions on Affective Computing. 2010;1(1):18–37.

[57] Bradley MM, Lang PJ. Affective Norms for English Words (ANEW): Instruction manual and affective ratings The Center for Research in Psychophysiology: University of Florida, 1999.

[58] Dodds P, Danforth C. Measuring the happiness of large-scale written expression: Songs, blogs, and presidents. J Happiness Stud. 2010;11(4):441–56.

[59] Owsley S, Sood S, Hammond KJ, editors. Domain specific affective classification of documents. AAAI Spring Symposium: Computational Approaches to Analyzing Weblogs; 2006.

[60] Asur S, Huberman BA. Predicting the future with social media. arXiv preprint arXiv:10035699. 2010.

[61] Johnson SL, Safadi H, Faraj S. The emergence of online community leadership. Information Systems Research. 2015;26(1):165–87.

[62] Willnat L, Zhu JH. Newspaper coverage and public opinion in Hong Kong: A time‐series analysis of media priming. Political Communication. 1996;13(2):231–46.

[63] Henningsen A, Hamann JD. Systemfit: A package for estimating systems of simultaneous equations in R. Journal of Statistical Software. 2007;23(4):1–40.

[64] Zellner A. An efficient method of estimating seemingly unrelated regressions and tests for aggregation bias. Journal of the American Statistical Association. 1962;57(298):348–68.

[65] Granger CWJ. Investigating Causal Relations by Econometric Models and Cross-spectral Methods. Econometrica. 1969;37(3):424–38.

[66] McElroy MB. Goodness of fit for seemingly unrelated regressions. Journal of Econometrics. 1977;6(3):381–7.

[67] Cohen J, Cohen P, West SG, Aiken LS. Applied multiple regression/correlation analysis for the behavioral sciences 3rd ed. Mahwah, NJ: Erlbaum; 2003.

[68] Durbin J, Watson GS. Testing for serial correlation in least squares regression: I. Biometrika. 1950;37(3/4):409–28. 14801065

[69] Kinder DR. Reason and emotion in American political life In: Schank RC, Lange E, editors. Beliefs, Reasoning, and Decision Making: Psycho-Logic in Honor of Bob Abelson. Hillsdale, New Jersey: Lawrence Erlbaum Associate 1994 p. 277–314.

[70] Scherer KR, Tannenbaum PH. Emotional experiences in everyday life: A survey approach. Motivation and Emotion. 1986;10(4):295–314.

[71] Huddy L, Gunnthorsdottir AH. The persuasive effects of emotive visual imagery: Superficial manipulation or the product of passionate reason? Political Psychology. 2000;21(4):745–78.

[72] Judd CM, McClelland GH, Culhane SE. Data analysis—Continuing issues in the everyday analysis of psychological data. Annual Review of Psychology. 1995;46:433–65. 10.1146/annurev.ps.46.020195.002245 7872734

[73] Robinson WS. Ecological correlations and the behavior of individuals. Americal Sociological Review. 1950;15:351–7.

[74] Xu X-K, Zhu JJH. Flexible sampling large-scale social networks by self-adjustable random walk. Physica A: Statistical Mechanics and its Applications. 2016;463:356–65.

[75] Ruths D, Pfeffer J. Social media for large studies of behavior. Science. 2014;346(6213):1063 10.1126/science.346.6213.1063 25430759

