# Supplementary material for: Interplay between Public Attention and Public Emotion toward Multiple Social Issues on Twitter
Source: PLoS One. 2017 Jan 12;12(1):e0167896. doi: 10.1371/journal.pone.0167896 (PMC5231282; doi:10.1371/journal.pone.0167896)
Supplement: S1 Table — (DOCX) [file pone.0167896.s001.docx]

S1 Table. List of Keywords on Five Issues

We adopted a bottom-up approach developed in Peng & Qin (2016) to generate the keywords used to retrieve tweets from Twitter Firehose. The bottom up approach will start with a set of seed keywords derived from the original codes of the Most Important Problems (MIP) questions in Gallup opinion polls. Each issue will have a list of seed keywords. Then the seed keywords of each issue will be submitted to Google Correlate, a product of Google, to get a longer list of keywords. The authors manually coded the keywords returned from Google Correlate to identify valid keywords for each issue. Finally, we obtained 111 valid keywords on the Economy issue, 983 valid keywords on the Government-Politics issue, 286 keywords on the Health issue, 238 keywords on the Job-Employment issue, and 564 keywords on the Money-Spending issue. The specific keywords for each issue are summarized in the table below.

| Issue | Keyword |
| --- | --- |
| Economy | 2008 recession |
| Economy | america economy |
| Economy | american economy |
| Economy | american recession |
| Economy | bad economy |
| Economy | banking system |
| Economy | china trade |
| Economy | cnn money market |
| Economy | consumer spending |
| Economy | current economic crisis |
| Economy | depression recession |
| Economy | depression wiki |
| Economy | depression? |
| Economy | difference between recession and depression |
| Economy | domestic product |
| Economy | down economy |
| Economy | economic |
| Economy | economic |
| Economy | economic condition |
| Economy | economic conditions |
| Economy | economic crisis |
| Economy | economic crisis in america |
| Economy | economic downturn |
| Economy | economic issues |
| Economy | economic news |
| Economy | economic policies |
| Economy | economic problems |
| Economy | economic recession |
| Economy | economic situation |
| Economy | economic state |
| Economy | economic statistics |
| Economy | economic status |
| Economy | economy |
| Economy | economy |
| Economy | economy problems |
| Economy | effect of recession |
| Economy | effects of recession |
| Economy | exports |
| Economy | federal reserve system |
| Economy | foreign trade |
| Economy | gdp 2008 |
| Economy | global economy |
| Economy | gnp |
| Economy | google finance dow |
| Economy | government economy |
| Economy | great depression |
| Economy | great depressions |
| Economy | gross domestic |
| Economy | gross domestic product |
| Economy | history of recessions |
| Economy | inflation |
| Economy | inflation rate |
| Economy | international trade |
| Economy | monetary system |
| Economy | our economy |
| Economy | past recessions |
| Economy | poor economy |
| Economy | recession |
| Economy | recession 2008 |
| Economy | recession and depression |
| Economy | recession chart |
| Economy | recession depression |
| Economy | recession economy |
| Economy | recession history |
| Economy | recession in the us |
| Economy | recession in us |
| Economy | recession in usa |
| Economy | recession news |
| Economy | recession or depression |
| Economy | recession periods |
| Economy | recession proof |
| Economy | recession proof business |
| Economy | recession proof businesses |
| Economy | recession proof careers |
| Economy | recession proof industries |
| Economy | recession proof jobs |
| Economy | recession us |
| Economy | recession years |
| Economy | recession? |
| Economy | recessions |
| Economy | state of the economy |
| Economy | states gdp |
| Economy | the current economy |
| Economy | the depression |
| Economy | the economic crisis |
| Economy | the economy |
| Economy | the great depression |
| Economy | the u.s. economy |
| Economy | the united states economy |
| Economy | the us economy |
| Economy | tough economic times |
| Economy | tough economy |
| Economy | trade deficit |
| Economy | u.s economy |
| Economy | u.s. economy |
| Economy | u.s. gdp |
| Economy | u.s. recession |
| Economy | u.s. recessions |
| Economy | u.s. trade deficit |
| Economy | united states economy |
| Economy | united states gdp |
| Economy | united states recession |
| Economy | us economic |
| Economy | us economy |
| Economy | us gdp 2008 |
| Economy | us recession |
| Economy | us recession history |
| Economy | us recessions |
| Economy | us trade deficit |
| Economy | world economic crisis |
| Economy | wsj libor |
| Government-Politics | "obama |
| Government-Politics | 0bama |
| Government-Politics | 2002 election |
| Government-Politics | 2004 campaign |
| Government-Politics | 2012 election |
| Government-Politics | 2012 electoral map |
| Government-Politics | 2012 polls |
| Government-Politics | 2012 presidential election |
| Government-Politics | 2012 presidential polls |
| Government-Politics | 2013 predictions |
| Government-Politics | 44 presidents of the united states |
| Government-Politics | about barack obama |
| Government-Politics | about earthquake |
| Government-Politics | about george bush |
| Government-Politics | about mitt romney |
| Government-Politics | about obama |
| Government-Politics | absentee votes |
| Government-Politics | abuse of power |
| Government-Politics | acorn voter |
| Government-Politics | aftershocks |
| Government-Politics | all hurricanes |
| Government-Politics | american election |
| Government-Politics | american fault lines |
| Government-Politics | american politics |
| Government-Politics | an earthquake |
| Government-Politics | anna eshoo |
| Government-Politics | anthony kennedy |
| Government-Politics | anthony m. kennedy |
| Government-Politics | anti bush |
| Government-Politics | anti bush shirts |
| Government-Politics | anti george bush |
| Government-Politics | anti-bush |
| Government-Politics | antonin scalia |
| Government-Politics | arizona ballot |
| Government-Politics | arizona elections |
| Government-Politics | arizona proposition |
| Government-Politics | arizona propositions |
| Government-Politics | arizona vote |
| Government-Politics | arizona voting |
| Government-Politics | atlantic hurricanes |
| Government-Politics | ayers and obama |
| Government-Politics | ayers obama |
| Government-Politics | bad bush |
| Government-Politics | ballot initiative |
| Government-Politics | ballot initiatives |
| Government-Politics | barack |
| Government-Politics | barack h obama |
| Government-Politics | barack obama |
| Government-Politics | barack obama autobiography |
| Government-Politics | barack obama biography |
| Government-Politics | barack obama change |
| Government-Politics | barack obama college |
| Government-Politics | barack obama funny |
| Government-Politics | barack obama hope |
| Government-Politics | barack obama images |
| Government-Politics | barack obama is |
| Government-Politics | barack obama name |
| Government-Politics | barack obama net worth |
| Government-Politics | barack obama pics |
| Government-Politics | barack obama quote |
| Government-Politics | barack obama quotes |
| Government-Politics | barack obama sr |
| Government-Politics | barack obama sr. |
| Government-Politics | barack obama white house |
| Government-Politics | barack obama wiki |
| Government-Politics | biggert |
| Government-Politics | biggest hurricane |
| Government-Politics | biggest hurricane ever |
| Government-Politics | biggest hurricanes |
| Government-Politics | bill ayer |
| Government-Politics | bill ayers |
| Government-Politics | bill ayers obama |
| Government-Politics | biography of barack obama |
| Government-Politics | biography of obama |
| Government-Politics | black remix |
| Government-Politics | blog obama |
| Government-Politics | blumenauer |
| Government-Politics | books by barack obama |
| Government-Politics | bsuh |
| Government-Politics | bush |
| Government-Politics | bush bad |
| Government-Politics | bush bio |
| Government-Politics | bush biography |
| Government-Politics | bush cartoons |
| Government-Politics | bush comics |
| Government-Politics | bush education |
| Government-Politics | bush environment |
| Government-Politics | bush family |
| Government-Politics | bush funny |
| Government-Politics | bush george |
| Government-Politics | bush history |
| Government-Politics | bush humor |
| Government-Politics | bush images |
| Government-Politics | bush is an idiot |
| Government-Politics | bush is stupid |
| Government-Politics | bush jokes |
| Government-Politics | bush official |
| Government-Politics | bush official site |
| Government-Politics | bush official website |
| Government-Politics | bush photo |
| Government-Politics | bush photos |
| Government-Politics | bush pics |
| Government-Politics | bush pictures |
| Government-Politics | bush policies |
| Government-Politics | bush policy |
| Government-Politics | bush posters |
| Government-Politics | bush president |
| Government-Politics | bush quotes |
| Government-Politics | bush sayings |
| Government-Politics | bush shirt |
| Government-Politics | bush shirts |
| Government-Politics | bush stupid |
| Government-Politics | bush support |
| Government-Politics | bush t-shirts |
| Government-Politics | bush w |
| Government-Politics | bush wallpaper |
| Government-Politics | bush website |
| Government-Politics | bush.com |
| Government-Politics | bush\ |
| Government-Politics | california congress |
| Government-Politics | california general election |
| Government-Politics | california representatives |
| Government-Politics | california secretary |
| Government-Politics | california senators |
| Government-Politics | campaign 2012 |
| Government-Politics | category 5 hurricane |
| Government-Politics | cause of earthquake |
| Government-Politics | center hurricane |
| Government-Politics | change we can believe in |
| Government-Politics | charles keating |
| Government-Politics | civil liberties |
| Government-Politics | close guantanamo bay |
| Government-Politics | cnn earthquake |
| Government-Politics | colorado election |
| Government-Politics | colorado elections |
| Government-Politics | colorado representatives |
| Government-Politics | colorado secretary |
| Government-Politics | colorado secretary of state |
| Government-Politics | colorado senators |
| Government-Politics | commissioners |
| Government-Politics | congres |
| Government-Politics | congress |
| Government-Politics | congress |
| Government-Politics | congress bills |
| Government-Politics | congress contact |
| Government-Politics | congress districts |
| Government-Politics | congress election |
| Government-Politics | congress elections |
| Government-Politics | congress email |
| Government-Politics | congress email address |
| Government-Politics | congress email addresses |
| Government-Politics | congress history |
| Government-Politics | congress house |
| Government-Politics | congress members |
| Government-Politics | congress phone numbers |
| Government-Politics | congress representative |
| Government-Politics | congress representatives |
| Government-Politics | congress senate |
| Government-Politics | congress website |
| Government-Politics | congress.gov |
| Government-Politics | congress.org |
| Government-Politics | congressional |
| Government-Politics | congressional committee |
| Government-Politics | congressional delegation |
| Government-Politics | congressional district |
| Government-Politics | congressional district map |
| Government-Politics | congressional districts |
| Government-Politics | congressional election |
| Government-Politics | congressional elections |
| Government-Politics | congressional email |
| Government-Politics | congressional email addresses |
| Government-Politics | congressional members |
| Government-Politics | congressional office |
| Government-Politics | congressional representative |
| Government-Politics | congressional representatives |
| Government-Politics | congressman |
| Government-Politics | congressman |
| Government-Politics | congressmen |
| Government-Politics | congressmen |
| Government-Politics | congressperson |
| Government-Politics | contact congress |
| Government-Politics | contact congressman |
| Government-Politics | contact congressmen |
| Government-Politics | contact my congressman |
| Government-Politics | contact representatives |
| Government-Politics | contact your congressman |
| Government-Politics | corruption |
| Government-Politics | court |
| Government-Politics | court building |
| Government-Politics | court cases |
| Government-Politics | court decision |
| Government-Politics | court decisions |
| Government-Politics | court judges |
| Government-Politics | court justice |
| Government-Politics | court justices |
| Government-Politics | court news |
| Government-Politics | court opinion |
| Government-Politics | court opinions |
| Government-Politics | court ruling |
| Government-Politics | court rulings |
| Government-Politics | court watch |
| Government-Politics | court website |
| Government-Politics | current hurricane |
| Government-Politics | current supreme court |
| Government-Politics | current supreme court justices |
| Government-Politics | dc electoral votes |
| Government-Politics | democratic senate |
| Government-Politics | democratic states |
| Government-Politics | dennis moore |
| Government-Politics | discurso de obama |
| Government-Politics | dubya |
| Government-Politics | dumb bush |
| Government-Politics | earthq |
| Government-Politics | earthquake |
| Government-Politics | earthquake 1989 |
| Government-Politics | earthquake activity map |
| Government-Politics | earthquake aftermath |
| Government-Politics | earthquake cause |
| Government-Politics | earthquake chart |
| Government-Politics | earthquake cnn |
| Government-Politics | earthquake coverage |
| Government-Politics | earthquake damage |
| Government-Politics | earthquake epicenter |
| Government-Politics | earthquake fault |
| Government-Politics | earthquake fault lines |
| Government-Politics | earthquake fault lines map |
| Government-Politics | earthquake history |
| Government-Politics | earthquake in |
| Government-Politics | earthquake in us |
| Government-Politics | earthquake jokes |
| Government-Politics | earthquake list |
| Government-Politics | earthquake magnitude |
| Government-Politics | earthquake magnitudes |
| Government-Politics | earthquake map |
| Government-Politics | earthquake measurement |
| Government-Politics | earthquake quotes |
| Government-Politics | earthquake rating |
| Government-Politics | earthquake richter scale |
| Government-Politics | earthquake risk |
| Government-Politics | earthquake scale |
| Government-Politics | earthquake size |
| Government-Politics | earthquake today |
| Government-Politics | earthquake today? |
| Government-Politics | earthquake twitter |
| Government-Politics | earthquake us |
| Government-Politics | earthquake youtube |
| Government-Politics | earthquake? |
| Government-Politics | earthquake. |
| Government-Politics | earthquake.usgs.gov |
| Government-Politics | earthquake\ |
| Government-Politics | earthquakes history |
| Government-Politics | earthquakes in us |
| Government-Politics | earthquakes this week |
| Government-Politics | elected officials |
| Government-Politics | election 2002 |
| Government-Politics | election 2012 |
| Government-Politics | election by state |
| Government-Politics | election date 2012 |
| Government-Politics | election day 2012 |
| Government-Politics | election history |
| Government-Politics | election jokes |
| Government-Politics | election poll results |
| Government-Politics | election prediction |
| Government-Politics | election president |
| Government-Politics | election presidential |
| Government-Politics | election race |
| Government-Politics | election reform |
| Government-Politics | election states |
| Government-Politics | election us |
| Government-Politics | election vote |
| Government-Politics | election voting |
| Government-Politics | electoral |
| Government-Politics | electoral college |
| Government-Politics | electoral college history |
| Government-Politics | electoral college map |
| Government-Politics | electoral college members |
| Government-Politics | electoral college results |
| Government-Politics | electoral college vote |
| Government-Politics | electoral college votes |
| Government-Politics | electoral college votes by state |
| Government-Politics | electoral college voting |
| Government-Politics | electoral colleges |
| Government-Politics | electoral count |
| Government-Politics | electoral states |
| Government-Politics | electoral vote |
| Government-Politics | electoral vote count |
| Government-Politics | electoral vote map |
| Government-Politics | electoral votes |
| Government-Politics | electoral votes map |
| Government-Politics | electoral voting |
| Government-Politics | electorate |
| Government-Politics | email congress |
| Government-Politics | email congressman |
| Government-Politics | email your congressman |
| Government-Politics | emergency management |
| Government-Politics | eshoo |
| Government-Politics | eye hurricane |
| Government-Politics | eye of a hurricane |
| Government-Politics | eye of hurricane |
| Government-Politics | facts about obama |
| Government-Politics | fault line |
| Government-Politics | fault line map |
| Government-Politics | fault lines |
| Government-Politics | fault lines in the us |
| Government-Politics | fault lines in us |
| Government-Politics | fault lines map |
| Government-Politics | federal house |
| Government-Politics | federal representatives |
| Government-Politics | federal senators |
| Government-Politics | federal supreme court |
| Government-Politics | find my congressman |
| Government-Politics | find your congressman |
| Government-Politics | find your representative |
| Government-Politics | florida electoral votes |
| Government-Politics | florida representatives |
| Government-Politics | florida secretary |
| Government-Politics | florida secretary of state |
| Government-Politics | florida senators |
| Government-Politics | for obama |
| Government-Politics | fundraisers |
| Government-Politics | fundraising |
| Government-Politics | funny barack obama |
| Government-Politics | funny bush |
| Government-Politics | funny bush pictures |
| Government-Politics | funny bush quotes |
| Government-Politics | funny george bush |
| Government-Politics | g w bush |
| Government-Politics | gag rule |
| Government-Politics | george bush |
| Government-Politics | george bush bio |
| Government-Politics | george bush biography |
| Government-Politics | george bush cartoons |
| Government-Politics | george bush family |
| Government-Politics | george bush jokes |
| Government-Politics | george bush photo |
| Government-Politics | george bush photos |
| Government-Politics | george bush pics |
| Government-Politics | george bush picture |
| Government-Politics | george bush pictures |
| Government-Politics | george bush quotes |
| Government-Politics | george w |
| Government-Politics | george w bush |
| Government-Politics | george w bush bio |
| Government-Politics | george w. |
| Government-Politics | george w. bush |
| Government-Politics | george w. bush bio |
| Government-Politics | george w. bush biography |
| Government-Politics | george w. bush quotes |
| Government-Politics | georgia representatives |
| Government-Politics | georgia senators |
| Government-Politics | google earthquake |
| Government-Politics | government branches |
| Government-Politics | government representatives |
| Government-Politics | government works |
| Government-Politics | guantanamo bay obama |
| Government-Politics | haarp earthquake |
| Government-Politics | hate obama |
| Government-Politics | health law |
| Government-Politics | help america vote |
| Government-Politics | help america vote act |
| Government-Politics | highest earthquake |
| Government-Politics | history of earthquakes |
| Government-Politics | history of hurricanes |
| Government-Politics | history of obama |
| Government-Politics | history of the supreme court |
| Government-Politics | house .gov |
| Government-Politics | house and senate |
| Government-Politics | house district |
| Government-Politics | house districts |
| Government-Politics | house election |
| Government-Politics | house elections |
| Government-Politics | house members |
| Government-Politics | house of rep |
| Government-Politics | house of representative |
| Government-Politics | house of representatives |
| Government-Politics | house of representatives email |
| Government-Politics | house of representatives email addresses |
| Government-Politics | house of representatives members |
| Government-Politics | house of reps |
| Government-Politics | house race |
| Government-Politics | house rep |
| Government-Politics | house representative |
| Government-Politics | house representatives |
| Government-Politics | house senate |
| Government-Politics | how does the electoral college work? |
| Government-Politics | how electoral college works |
| Government-Politics | how the electoral college works |
| Government-Politics | hurric |
| Government-Politics | hurricane |
| Government-Politics | hurricane atlantic |
| Government-Politics | hurricane carol |
| Government-Politics | hurricane center |
| Government-Politics | hurricane conditions |
| Government-Politics | hurricane david |
| Government-Politics | hurricane diana |
| Government-Politics | hurricane diane |
| Government-Politics | hurricane eye |
| Government-Politics | hurricane flag |
| Government-Politics | hurricane flags |
| Government-Politics | hurricane hazel |
| Government-Politics | hurricane history |
| Government-Politics | hurricane hugo |
| Government-Politics | hurricane hunter |
| Government-Politics | hurricane hunters |
| Government-Politics | hurricane info |
| Government-Politics | hurricane landfall |
| Government-Politics | hurricane list |
| Government-Politics | hurricane map |
| Government-Politics | hurricane name |
| Government-Politics | hurricane names |
| Government-Politics | hurricane national |
| Government-Politics | hurricane news |
| Government-Politics | hurricane noaa |
| Government-Politics | hurricane radar |
| Government-Politics | hurricane report |
| Government-Politics | hurricane rotation |
| Government-Politics | hurricane satellite |
| Government-Politics | hurricane shelter |
| Government-Politics | hurricane size |
| Government-Politics | hurricane status |
| Government-Politics | hurricane storm |
| Government-Politics | hurricane storm surge |
| Government-Politics | hurricane track |
| Government-Politics | hurricane update |
| Government-Politics | hurricane updates |
| Government-Politics | hurricane warning |
| Government-Politics | hurricane watch |
| Government-Politics | hurricane wind |
| Government-Politics | hurricane wind speed |
| Government-Politics | hurricane winds |
| Government-Politics | hurricane\ |
| Government-Politics | hurricanes |
| Government-Politics | hurricanes by year |
| Government-Politics | hurricanes history |
| Government-Politics | hurricanes in history |
| Government-Politics | hurricanes names |
| Government-Politics | hurrikan |
| Government-Politics | i hate obama |
| Government-Politics | illinois senators |
| Government-Politics | immigrating |
| Government-Politics | inside of the white house |
| Government-Politics | interactive electoral map |
| Government-Politics | interactive hurricane |
| Government-Politics | interest group |
| Government-Politics | interest groups |
| Government-Politics | is obama |
| Government-Politics | jay z my president is black |
| Government-Politics | jewish vote |
| Government-Politics | judicial system |
| Government-Politics | judy biggert |
| Government-Politics | justices |
| Government-Politics | justices of the supreme court |
| Government-Politics | justices supreme court |
| Government-Politics | landfall |
| Government-Politics | largest earthquake in us |
| Government-Politics | largest hurricane |
| Government-Politics | last earthquake |
| Government-Politics | latest earthquake news |
| Government-Politics | latest hurricane |
| Government-Politics | law and justice |
| Government-Politics | league of women voters minnesota |
| Government-Politics | legal system |
| Government-Politics | letters to obama |
| Government-Politics | limbaugh obama |
| Government-Politics | list of congressmen |
| Government-Politics | list of earthquakes |
| Government-Politics | list of hurricane names |
| Government-Politics | list of hurricanes |
| Government-Politics | list of senators |
| Government-Politics | live hurricane |
| Government-Politics | ma vote |
| Government-Politics | ma voting |
| Government-Politics | magnitude and intensity |
| Government-Politics | magnitude earthquake |
| Government-Politics | magnitude of earthquake |
| Government-Politics | major fault lines |
| Government-Politics | major hurricanes |
| Government-Politics | malia and sasha |
| Government-Politics | malia and sasha obama |
| Government-Politics | malia sasha |
| Government-Politics | map of electoral votes |
| Government-Politics | map of fault lines |
| Government-Politics | mass elections |
| Government-Politics | massachusetts ballot |
| Government-Politics | massachusetts elections |
| Government-Politics | massachusetts voting |
| Government-Politics | members of supreme court |
| Government-Politics | members of the supreme court |
| Government-Politics | michelle obama dresses |
| Government-Politics | michigan senators |
| Government-Politics | minnesota election |
| Government-Politics | minnesota elections |
| Government-Politics | minnesota secretary of state |
| Government-Politics | missouri senators |
| Government-Politics | mitt romney |
| Government-Politics | mitt romney campaign |
| Government-Politics | mitt romney governor |
| Government-Politics | mitt romney massachusetts |
| Government-Politics | mitt romney polls |
| Government-Politics | mitt romney quotes |
| Government-Politics | mitt romney website |
| Government-Politics | mittromney.com |
| Government-Politics | mn secretary of state |
| Government-Politics | mn voting |
| Government-Politics | most powerful hurricane |
| Government-Politics | my congressional representative |
| Government-Politics | my congressman |
| Government-Politics | my president |
| Government-Politics | my president is black |
| Government-Politics | my president is black jay z |
| Government-Politics | my president is black remix |
| Government-Politics | my representative |
| Government-Politics | my representative in congress |
| Government-Politics | my representatives |
| Government-Politics | my senator |
| Government-Politics | names of hurricanes |
| Government-Politics | nasa hurricane |
| Government-Politics | national earthquake center |
| Government-Politics | national geological survey |
| Government-Politics | national hurricane center |
| Government-Politics | nevada voting |
| Government-Politics | new jersey senators |
| Government-Politics | new york state election |
| Government-Politics | new york state elections |
| Government-Politics | new york times election |
| Government-Politics | news about obama |
| Government-Politics | news earthquake |
| Government-Politics | news hurricane |
| Government-Politics | news on earthquake |
| Government-Politics | news on obama |
| Government-Politics | nj congressman |
| Government-Politics | nj senators |
| Government-Politics | no bush |
| Government-Politics | noaa earthquake |
| Government-Politics | noaa national hurricane center |
| Government-Politics | north american fault lines |
| Government-Politics | northridge earthquake facts |
| Government-Politics | number of electoral votes |
| Government-Politics | obama |
| Government-Politics | obama .com |
| Government-Politics | obama a |
| Government-Politics | obama and |
| Government-Politics | obama and abortion |
| Government-Politics | obama and lincoln |
| Government-Politics | obama apparel |
| Government-Politics | obama astrology |
| Government-Politics | obama autobiography |
| Government-Politics | obama ayers |
| Government-Politics | obama bad |
| Government-Politics | obama bible |
| Government-Politics | obama bill ayers |
| Government-Politics | obama biography |
| Government-Politics | obama bobble head |
| Government-Politics | obama books |
| Government-Politics | obama change |
| Government-Politics | obama cnn |
| Government-Politics | obama coloring pages |
| Government-Politics | obama con |
| Government-Politics | obama date of birth |
| Government-Politics | obama facts |
| Government-Politics | obama father |
| Government-Politics | obama funny |
| Government-Politics | obama graphic |
| Government-Politics | obama guantanamo bay |
| Government-Politics | obama history |
| Government-Politics | obama horoscope |
| Government-Politics | obama humor |
| Government-Politics | obama ideas |
| Government-Politics | obama images |
| Government-Politics | obama in 2008 |
| Government-Politics | obama in the white house |
| Government-Politics | obama is |
| Government-Politics | obama is bad |
| Government-Politics | obama is evil |
| Government-Politics | obama life |
| Government-Politics | obama logo |
| Government-Politics | obama memorabilia |
| Government-Politics | obama mother |
| Government-Politics | obama name |
| Government-Politics | obama net worth |
| Government-Politics | obama obey |
| Government-Politics | obama pics |
| Government-Politics | obama plans |
| Government-Politics | obama poem |
| Government-Politics | obama policies |
| Government-Politics | obama policy |
| Government-Politics | obama posters |
| Government-Politics | obama quotes |
| Government-Politics | obama service |
| Government-Politics | obama speeches |
| Government-Politics | obama story |
| Government-Politics | obama videos |
| Government-Politics | obama website |
| Government-Politics | obama white house staff |
| Government-Politics | obama wiki |
| Government-Politics | obama wikipedia |
| Government-Politics | obama youtube |
| Government-Politics | obama? |
| Government-Politics | obama. |
| Government-Politics | obama.org |
| Government-Politics | obama\ |
| Government-Politics | obamarama |
| Government-Politics | obey obama |
| Government-Politics | office of the president of the united states |
| Government-Politics | official white house website |
| Government-Politics | ohio representatives |
| Government-Politics | ohio senator |
| Government-Politics | ohio senators |
| Government-Politics | oregon elections |
| Government-Politics | oregon representatives |
| Government-Politics | oregon senators |
| Government-Politics | oregon voting |
| Government-Politics | past election results |
| Government-Politics | past elections |
| Government-Politics | past hurricanes |
| Government-Politics | past presidential elections |
| Government-Politics | path of hurricane |
| Government-Politics | photos of white house |
| Government-Politics | picture of hurricane |
| Government-Politics | political action |
| Government-Politics | political corruption |
| Government-Politics | poll 2012 |
| Government-Politics | poll romney |
| Government-Politics | polls 2012 |
| Government-Politics | polls bush |
| Government-Politics | polls election |
| Government-Politics | president bush |
| Government-Politics | president george w bush |
| Government-Politics | president george w. bush |
| Government-Politics | president is black |
| Government-Politics | president obama cabinet |
| Government-Politics | president obama family |
| Government-Politics | president obama inauguration |
| Government-Politics | president race |
| Government-Politics | president vote |
| Government-Politics | president votes |
| Government-Politics | presidente barack obama |
| Government-Politics | presidential ball |
| Government-Politics | presidential election |
| Government-Politics | presidential election 2012 |
| Government-Politics | presidential election history |
| Government-Politics | presidential election map |
| Government-Politics | presidential election maps |
| Government-Politics | presidential election news |
| Government-Politics | presidential election voting |
| Government-Politics | presidential elections |
| Government-Politics | presidential elections history |
| Government-Politics | presidential electoral votes |
| Government-Politics | presidential race |
| Government-Politics | presidential races |
| Government-Politics | presidential salary |
| Government-Politics | presidential vote |
| Government-Politics | presidential voting |
| Government-Politics | pro bush |
| Government-Politics | pro-bush |
| Government-Politics | problems with obama |
| Government-Politics | property rights |
| Government-Politics | public service commission |
| Government-Politics | purpose of electoral college |
| Government-Politics | quotes from barack obama |
| Government-Politics | quotes from obama |
| Government-Politics | referendums |
| Government-Politics | registered voters |
| Government-Politics | religious right |
| Government-Politics | representatives |
| Government-Politics | representatives in congress |
| Government-Politics | republican bush |
| Government-Politics | republican congressman |
| Government-Politics | richter scale |
| Government-Politics | rob bishop |
| Government-Politics | romey |
| Government-Politics | romne |
| Government-Politics | romney |
| Government-Politics | romney campaign |
| Government-Politics | romney governor |
| Government-Politics | romney height |
| Government-Politics | romney issues |
| Government-Politics | romney massachusetts |
| Government-Politics | romney on abortion |
| Government-Politics | romney platform |
| Government-Politics | romney poll |
| Government-Politics | romney polls |
| Government-Politics | romney quotes |
| Government-Politics | romney website |
| Government-Politics | rooms in the white house |
| Government-Politics | sam farr |
| Government-Politics | sasha and malia |
| Government-Politics | sasha and malia obama |
| Government-Politics | sasha malia |
| Government-Politics | sasha obama |
| Government-Politics | satellite hurricane |
| Government-Politics | scalia |
| Government-Politics | seattle earthquake 2001 |
| Government-Politics | secretary of state |
| Government-Politics | seismic activity |
| Government-Politics | seismic scale |
| Government-Politics | sen. |
| Government-Politics | senat |
| Government-Politics | senate |
| Government-Politics | senate and house |
| Government-Politics | senate breakdown |
| Government-Politics | senate congress |
| Government-Politics | senate districts |
| Government-Politics | senate election |
| Government-Politics | senate election map |
| Government-Politics | senate election results |
| Government-Politics | senate elections |
| Government-Politics | senate house |
| Government-Politics | senate makeup |
| Government-Politics | senate map |
| Government-Politics | senate numbers |
| Government-Politics | senate race |
| Government-Politics | senate races |
| Government-Politics | senate representatives |
| Government-Politics | senate results |
| Government-Politics | senate seats |
| Government-Politics | senate term |
| Government-Politics | senate terms |
| Government-Politics | senate us |
| Government-Politics | senate website |
| Government-Politics | senate.gov |
| Government-Politics | senates |
| Government-Politics | senator |
| Government-Politics | senator list |
| Government-Politics | senatorial |
| Government-Politics | senatorial elections |
| Government-Politics | senators |
| Government-Politics | state assembly |
| Government-Politics | state board of elections |
| Government-Politics | state congress |
| Government-Politics | state congressional districts |
| Government-Politics | state congressman |
| Government-Politics | state constitution |
| Government-Politics | state elections |
| Government-Politics | state electoral votes |
| Government-Politics | state house |
| Government-Politics | state house of representatives |
| Government-Politics | state representative |
| Government-Politics | state representatives |
| Government-Politics | state senate |
| Government-Politics | state senator |
| Government-Politics | state senators |
| Government-Politics | state votes |
| Government-Politics | state website |
| Government-Politics | states electoral votes |
| Government-Politics | storm path |
| Government-Politics | storm track |
| Government-Politics | stupid bush |
| Government-Politics | stupid bush quotes |
| Government-Politics | support bush |
| Government-Politics | supreme court |
| Government-Politics | supreme court appointees |
| Government-Politics | supreme court appointments |
| Government-Politics | supreme court blog |
| Government-Politics | supreme court building |
| Government-Politics | supreme court decision |
| Government-Politics | supreme court decisions |
| Government-Politics | supreme court history |
| Government-Politics | supreme court judge |
| Government-Politics | supreme court judges |
| Government-Politics | supreme court justice |
| Government-Politics | supreme court justices |
| Government-Politics | supreme court members |
| Government-Politics | supreme court news |
| Government-Politics | supreme court of the united states |
| Government-Politics | supreme court of the us |
| Government-Politics | supreme court of united states |
| Government-Politics | supreme court of us |
| Government-Politics | supreme court opinion |
| Government-Politics | supreme court opinions |
| Government-Politics | supreme court rulings |
| Government-Politics | supreme court united states |
| Government-Politics | supreme court us |
| Government-Politics | supreme court vote |
| Government-Politics | supreme court votes |
| Government-Politics | supreme court watch |
| Government-Politics | supreme court website |
| Government-Politics | supreme courts |
| Government-Politics | supreme justice |
| Government-Politics | supreme justices |
| Government-Politics | tectonic plate map |
| Government-Politics | term limits |
| Government-Politics | texas congress |
| Government-Politics | texas representatives |
| Government-Politics | texas secretary of state |
| Government-Politics | texas senators |
| Government-Politics | texas us |
| Government-Politics | the 2012 election |
| Government-Politics | the bush family |
| Government-Politics | the earthquake |
| Government-Politics | the electoral college |
| Government-Politics | the national hurricane center |
| Government-Politics | the obama family |
| Government-Politics | the obama girls |
| Government-Politics | the president of the united states |
| Government-Politics | the supreme court justices |
| Government-Politics | the united states supreme court |
| Government-Politics | the us senate |
| Government-Politics | the us supreme court |
| Government-Politics | the white house |
| Government-Politics | the white house address |
| Government-Politics | the white house pictures |
| Government-Politics | the white house rooms |
| Government-Politics | the white house website |
| Government-Politics | thewhitehouse.gov |
| Government-Politics | tiberi |
| Government-Politics | today earthquake |
| Government-Politics | todd platts |
| Government-Politics | tour of the white house |
| Government-Politics | track hurricane |
| Government-Politics | track hurricanes |
| Government-Politics | tracking hurricane |
| Government-Politics | tracking hurricanes |
| Government-Politics | u s congress |
| Government-Politics | u s house |
| Government-Politics | u s house of representatives |
| Government-Politics | u s senate |
| Government-Politics | u s senators |
| Government-Politics | u s supreme court |
| Government-Politics | u. s. house of representatives |
| Government-Politics | u. s. senate |
| Government-Politics | u. s. senators |
| Government-Politics | u. s. supreme court |
| Government-Politics | u.s election |
| Government-Politics | u.s house |
| Government-Politics | u.s house of representatives |
| Government-Politics | u.s senate |
| Government-Politics | u.s supreme court |
| Government-Politics | u.s. congress |
| Government-Politics | u.s. congressmen |
| Government-Politics | u.s. house |
| Government-Politics | u.s. house of representatives |
| Government-Politics | u.s. representative |
| Government-Politics | u.s. representatives |
| Government-Politics | u.s. senate |
| Government-Politics | u.s. senators |
| Government-Politics | u.s. supreme court |
| Government-Politics | u.s. supreme court decisions |
| Government-Politics | u.s. supreme court justices |
| Government-Politics | u.s.senate |
| Government-Politics | u.s.supreme court |
| Government-Politics | united state senate |
| Government-Politics | united state supreme court |
| Government-Politics | united states congress |
| Government-Politics | united states electoral college |
| Government-Politics | united states fault lines |
| Government-Politics | united states house |
| Government-Politics | united states house of representatives |
| Government-Politics | united states presidential election |
| Government-Politics | united states senate |
| Government-Politics | united states senator |
| Government-Politics | united states senators |
| Government-Politics | united states supreme court |
| Government-Politics | united states supreme court justices |
| Government-Politics | us congress |
| Government-Politics | us congress email |
| Government-Politics | us congress members |
| Government-Politics | us congressman |
| Government-Politics | us congressmen |
| Government-Politics | us earthquake |
| Government-Politics | us earthquake map |
| Government-Politics | us earthquakes |
| Government-Politics | us election |
| Government-Politics | us electoral college |
| Government-Politics | us fault lines |
| Government-Politics | us government structure |
| Government-Politics | us house |
| Government-Politics | us house of rep |
| Government-Politics | us house of representative |
| Government-Politics | us house of representatives |
| Government-Politics | us house of representatives members |
| Government-Politics | us house of reps |
| Government-Politics | us house representative |
| Government-Politics | us house representatives |
| Government-Politics | us hurricane |
| Government-Politics | us hurricane center |
| Government-Politics | us political |
| Government-Politics | us presidential election |
| Government-Politics | us presidential elections |
| Government-Politics | us rep |
| Government-Politics | us representatives |
| Government-Politics | us senate |
| Government-Politics | us senate election |
| Government-Politics | us senate elections |
| Government-Politics | us senate race |
| Government-Politics | us senate seats |
| Government-Politics | us senate term |
| Government-Politics | us senate terms |
| Government-Politics | us senator |
| Government-Politics | us senators |
| Government-Politics | us supreme |
| Government-Politics | us supreme court |
| Government-Politics | us supreme court decision |
| Government-Politics | us supreme court decisions |
| Government-Politics | us supreme court justice |
| Government-Politics | us supreme court justices |
| Government-Politics | us supreme court members |
| Government-Politics | us voting |
| Government-Politics | usgs earthquake |
| Government-Politics | vermont elections |
| Government-Politics | video of earthquake |
| Government-Politics | virginia congressional districts |
| Government-Politics | virginia senators |
| Government-Politics | volokh |
| Government-Politics | vote counts |
| Government-Politics | vote map |
| Government-Politics | vote massachusetts |
| Government-Politics | vote president |
| Government-Politics | vote statistics |
| Government-Politics | vote tracker |
| Government-Politics | voting demographics |
| Government-Politics | voting district |
| Government-Politics | voting districts |
| Government-Politics | voting patterns |
| Government-Politics | voting systems |
| Government-Politics | w bush |
| Government-Politics | w. bush |
| Government-Politics | washington state senators |
| Government-Politics | weather hurricane |
| Government-Politics | what causes a earthquake |
| Government-Politics | what causes an earthquake |
| Government-Politics | what causes earthquakes |
| Government-Politics | what district am i in? |
| Government-Politics | what is a hurricane |
| Government-Politics | what is barack obama |
| Government-Politics | what is electoral college |
| Government-Politics | what is guantanamo bay |
| Government-Politics | what is hurricane |
| Government-Politics | what is the electoral college |
| Government-Politics | what size is michelle obama |
| Government-Politics | what to do during an earthquake |
| Government-Politics | what to do in an earthquake |
| Government-Politics | what to do in case of an earthquake |
| Government-Politics | when was the last earthquake |
| Government-Politics | where is barack obama |
| Government-Politics | where was the earthquake |
| Government-Politics | white house |
| Government-Politics | white house .gov |
| Government-Politics | white house address |
| Government-Politics | white house floor plan |
| Government-Politics | white house gov |
| Government-Politics | white house inside |
| Government-Politics | white house official website |
| Government-Politics | white house photos |
| Government-Politics | white house pictures |
| Government-Politics | white house rooms |
| Government-Politics | white house site |
| Government-Politics | white house staff |
| Government-Politics | white house.gov |
| Government-Politics | white house.org |
| Government-Politics | whitehouse |
| Government-Politics | whitehouse website |
| Government-Politics | whitehouse.gov |
| Government-Politics | whitehouse.gove |
| Government-Politics | who is mitt romney |
| Government-Politics | who is my congressman |
| Government-Politics | who is my congressman? |
| Government-Politics | who is my house representative |
| Government-Politics | who is my representative |
| Government-Politics | who is my representative in congress |
| Government-Politics | who is my senator |
| Government-Politics | who is obama |
| Government-Politics | who names hurricanes |
| Government-Politics | why electoral college |
| Government-Politics | wiki obama |
| Government-Politics | wikipedia obama |
| Government-Politics | william ayers |
| Government-Politics | william ayres |
| Government-Politics | worst hurricane |
| Government-Politics | worst hurricane ever |
| Government-Politics | worst hurricanes |
| Government-Politics | worst hurricanes in history |
| Government-Politics | write congressman |
| Government-Politics | write my congressman |
| Government-Politics | write your congressman |
| Government-Politics | www.bush.com |
| Government-Politics | www.hurricane |
| Government-Politics | www.obama |
| Government-Politics | www.supremecourt.gov |
| Government-Politics | youngest president of the united states |
| Government-Politics | youth vote |
| Government-Politics | youtube earthquake |
| Government-Politics | youtube obama |
| Health | 1918 flu epidemic |
| Health | 1918 influenza epidemic |
| Health | 2009 swine flu |
| Health | 3m 1860 |
| Health | 3m n95 |
| Health | about breast cancer |
| Health | about health care |
| Health | about swine flu |
| Health | against healthcare reform |
| Health | american health |
| Health | american health care |
| Health | antiviral |
| Health | average health insurance cost |
| Health | breast cancer |
| Health | breast cancer hope |
| Health | breast cancer pink ribbon |
| Health | breast cancer research |
| Health | breast cancer ribbons |
| Health | breast cancer statistics |
| Health | buy insurance |
| Health | cancer |
| Health | cancer facts |
| Health | cancer organizations |
| Health | cancer pink ribbon |
| Health | cancer statistics |
| Health | care costs |
| Health | care legislation |
| Health | cdc |
| Health | cdc flu shot |
| Health | cdc swine |
| Health | cdc swine flu |
| Health | cdc.gov |
| Health | child support guidelines |
| Health | cnn health care |
| Health | common flu |
| Health | cons of health care reform |
| Health | deaths from flu |
| Health | disability awareness |
| Health | doctor salary |
| Health | doctors salaries |
| Health | eligible for medicaid |
| Health | epidemic |
| Health | facts about health |
| Health | facts about health care |
| Health | fatty acid |
| Health | find a flu shot |
| Health | flu |
| Health | flu 1918 |
| Health | flu 2009 |
| Health | flu alert |
| Health | flu antiviral |
| Health | flu cases |
| Health | flu causes |
| Health | flu cdc |
| Health | flu cure |
| Health | flu death |
| Health | flu deaths |
| Health | flu definition |
| Health | flu diagnosis |
| Health | flu epidemic |
| Health | flu epidemic 1918 |
| Health | flu epidemics |
| Health | flu facts |
| Health | flu history |
| Health | flu info |
| Health | flu information |
| Health | flu map |
| Health | flu maps |
| Health | flu mask |
| Health | flu masks |
| Health | flu medication |
| Health | flu mortality rate |
| Health | flu new york |
| Health | flu news |
| Health | flu outbreak |
| Health | flu outbreak map |
| Health | flu outbreaks |
| Health | flu pandemic 1918 |
| Health | flu pandemics |
| Health | flu precautions |
| Health | flu reports |
| Health | flu signs |
| Health | flu signs and symptoms |
| Health | flu spread |
| Health | flu stats |
| Health | flu systems |
| Health | flu timeline |
| Health | flu transmission |
| Health | flu update |
| Health | flu vaccine shortage |
| Health | flu virus |
| Health | flu virus symptoms |
| Health | flu viruses |
| Health | flu wiki |
| Health | flu wikipedia |
| Health | fluenza |
| Health | fox news health |
| Health | free medical |
| Health | general health |
| Health | get health insurance |
| Health | government health care bill |
| Health | group health cooperative |
| Health | health |
| Health | health and |
| Health | health and human |
| Health | health benefits |
| Health | health bill |
| Health | health care |
| Health | health care bill |
| Health | health care bill cost |
| Health | health care bill facts |
| Health | health care bill passed |
| Health | health care bill pdf |
| Health | health care bill summary |
| Health | health care bill text |
| Health | health care cost |
| Health | health care costs |
| Health | health care coverage |
| Health | health care exchange |
| Health | health care insurance |
| Health | health care legislation |
| Health | health care overhaul |
| Health | health care plan |
| Health | health care plan summary |
| Health | health care poll |
| Health | health care polls |
| Health | health care reform |
| Health | health care reform bill |
| Health | health care reform bill passed |
| Health | health care reform cost |
| Health | health care reform facts |
| Health | health care reform for dummies |
| Health | health care reform medicare |
| Health | health care reform passed |
| Health | health care reform pros |
| Health | health care reform pros and cons |
| Health | health care reform summary |
| Health | health care reform wiki |
| Health | health care summary |
| Health | health care systems |
| Health | health care taxes |
| Health | health care vote |
| Health | health coverage |
| Health | health exchange |
| Health | health insurance |
| Health | health insurance |
| Health | health insurance bill |
| Health | health insurance company |
| Health | health insurance cost |
| Health | health insurance cost per month |
| Health | health insurance coverage |
| Health | health insurance exchange |
| Health | health insurance exchanges |
| Health | health plan |
| Health | health plans |
| Health | health program |
| Health | health reform |
| Health | health reform bill |
| Health | health services |
| Health | health-care |
| Health | hong kong flu |
| Health | how much does health insurance cost |
| Health | how much does health insurance cost per month |
| Health | how much is health insurance |
| Health | individual health |
| Health | infection control |
| Health | influenza |
| Health | influenza deaths |
| Health | influenza epidemic |
| Health | influenza outbreak |
| Health | influenza virus |
| Health | insurance agents |
| Health | insurance brokers |
| Health | insurance company |
| Health | insurance cost |
| Health | insurance costs |
| Health | insurance exchanges |
| Health | insurance laws |
| Health | kaiser family |
| Health | kaiser family foundation |
| Health | kaiser health news |
| Health | la influenza |
| Health | life insurance |
| Health | medicaid vs medicare |
| Health | medical devices |
| Health | medical masks |
| Health | medical reform |
| Health | medical transcription |
| Health | medicare 1965 |
| Health | medicare increase |
| Health | mental health |
| Health | mutual insurance |
| Health | mutual insurance company |
| Health | n95 mask |
| Health | national breast cancer |
| Health | national health |
| Health | national health care |
| Health | national healthcare |
| Health | new health |
| Health | new health bill |
| Health | new health care |
| Health | new health care reform |
| Health | new health care reform bill |
| Health | new health plan |
| Health | new healthcare bill |
| Health | new healthcare plan |
| Health | new healthcare reform |
| Health | new york times health care |
| Health | nixon health care |
| Health | nurse practitioners |
| Health | nyu medical |
| Health | obama health bill |
| Health | obama health care bill |
| Health | obama health care plan explained |
| Health | obama health care reform bill |
| Health | on health |
| Health | oncology |
| Health | oseltamivir |
| Health | pandemic 1918 |
| Health | pandemic history |
| Health | pandemics |
| Health | pathology |
| Health | poll health care |
| Health | pre existing condition |
| Health | pre existing conditions |
| Health | preventative care |
| Health | primary care physician |
| Health | private health |
| Health | pros and cons of health care |
| Health | pros and cons of health care reform |
| Health | pros and cons of healthcare |
| Health | prudential insurance |
| Health | public health |
| Health | quotes on health care |
| Health | reform health care |
| Health | reforma de salud |
| Health | rehabilitation |
| Health | republicans health care |
| Health | self insured |
| Health | sign health |
| Health | skin cancer |
| Health | spanish flu 1918 |
| Health | spanish flu epidemic |
| Health | spanish influenza |
| Health | summary of health care reform |
| Health | sun protection |
| Health | support health care reform |
| Health | surgical mask |
| Health | surgical masks |
| Health | swine flu |
| Health | swine flu cases |
| Health | swine flu death |
| Health | swine flu facts |
| Health | swine flu outbreak |
| Health | swine flu spread |
| Health | swine flu virus |
| Health | swine flu? |
| Health | symptoms of the swine flu |
| Health | tamiflu wiki |
| Health | term life insurance |
| Health | the health |
| Health | the health care bill |
| Health | the health care reform |
| Health | the health care reform bill |
| Health | the healthcare bill |
| Health | the swine flu |
| Health | u.s. health |
| Health | uninsured |
| Health | universal health care bill |
| Health | university medical |
| Health | us health |
| Health | us health care |
| Health | us health care bill |
| Health | us health care reform |
| Health | us healthcare |
| Health | us healthcare reform |
| Health | usa health |
| Health | wall street journal health care |
| Health | washington post health care |
| Health | what is health care |
| Health | what is health care reform |
| Health | what is medicaid |
| Health | what is the health care reform |
| Health | when did medicare start |
| Health | who flu |
| Health | zanamivir |
| Job-Employment | a job |
| Job-Employment | about unemployment |
| Job-Employment | accounting jobs |
| Job-Employment | administrator jobs |
| Job-Employment | airport jobs |
| Job-Employment | alabama unemployment |
| Job-Employment | apple jobs |
| Job-Employment | arizona jobs |
| Job-Employment | arkansas unemployment |
| Job-Employment | assistant jobs |
| Job-Employment | assistant manager |
| Job-Employment | assistant resume |
| Job-Employment | bank jobs |
| Job-Employment | beach jobs |
| Job-Employment | benefits for unemployed |
| Job-Employment | ca unemployment |
| Job-Employment | ca unemployment extension |
| Job-Employment | ca unemployment rate |
| Job-Employment | calculate unemployment benefits |
| Job-Employment | california unemployment rate |
| Job-Employment | care jobs |
| Job-Employment | careerbuilder.com jobseeker |
| Job-Employment | caribbean jobs |
| Job-Employment | carolina jobs |
| Job-Employment | census bureau employment |
| Job-Employment | colorado unemployment |
| Job-Employment | colorado unemployment rate |
| Job-Employment | company jobs |
| Job-Employment | company layoffs |
| Job-Employment | counseling jobs |
| Job-Employment | craigslist job |
| Job-Employment | current unemployment |
| Job-Employment | current unemployment rate |
| Job-Employment | desempleo |
| Job-Employment | does unemployment |
| Job-Employment | eligible for unemployment |
| Job-Employment | emergency unemployment |
| Job-Employment | emergency unemployment benefits |
| Job-Employment | emergency unemployment compensation |
| Job-Employment | employment |
| Job-Employment | employment agencies |
| Job-Employment | employment agency |
| Job-Employment | employment in |
| Job-Employment | employment services |
| Job-Employment | entertainment jobs |
| Job-Employment | express employment |
| Job-Employment | extended benefits |
| Job-Employment | extended unemployment |
| Job-Employment | extended unemployment benefits |
| Job-Employment | extension for unemployment |
| Job-Employment | extension for unemployment benefits |
| Job-Employment | extension on unemployment |
| Job-Employment | extension unemployment |
| Job-Employment | federal employment |
| Job-Employment | federal job |
| Job-Employment | federal jobs |
| Job-Employment | federal unemployment benefits |
| Job-Employment | file unemployment |
| Job-Employment | florida employment |
| Job-Employment | florida unemployment |
| Job-Employment | food service jobs |
| Job-Employment | for jobs |
| Job-Employment | ga dept of labor |
| Job-Employment | ga unemployment |
| Job-Employment | get a job |
| Job-Employment | get hired |
| Job-Employment | government job |
| Job-Employment | health aide |
| Job-Employment | healthcare jobs |
| Job-Employment | highest unemployment rate |
| Job-Employment | hired |
| Job-Employment | hiring |
| Job-Employment | hiring now |
| Job-Employment | home health aide |
| Job-Employment | hospital careers |
| Job-Employment | how long are unemployment benefits |
| Job-Employment | how long do unemployment benefits last |
| Job-Employment | how long is unemployment |
| Job-Employment | how long is unemployment benefits |
| Job-Employment | how long unemployment benefits |
| Job-Employment | how many weeks of unemployment |
| Job-Employment | how much are unemployment benefits |
| Job-Employment | how much do you make |
| Job-Employment | how much should i charge |
| Job-Employment | how to calculate unemployment benefits |
| Job-Employment | how to get a job |
| Job-Employment | human resources jobs |
| Job-Employment | il unemployment |
| Job-Employment | illinois unemployment |
| Job-Employment | in unemployment |
| Job-Employment | it job |
| Job-Employment | it jobs |
| Job-Employment | job board |
| Job-Employment | job cut |
| Job-Employment | job cuts |
| Job-Employment | job hiring |
| Job-Employment | job layoffs |
| Job-Employment | job listing |
| Job-Employment | job listings |
| Job-Employment | job opportunities |
| Job-Employment | job placement |
| Job-Employment | job quotes |
| Job-Employment | job search |
| Job-Employment | job service |
| Job-Employment | job sites |
| Job-Employment | jobless |
| Job-Employment | jobless claims |
| Job-Employment | jobless rate |
| Job-Employment | jobs |
| Job-Employment | jobs be |
| Job-Employment | jobs colorado |
| Job-Employment | jobs facebook |
| Job-Employment | jobs hiring |
| Job-Employment | jobs in |
| Job-Employment | jobs in apple |
| Job-Employment | jobs in austin |
| Job-Employment | jobs in colorado |
| Job-Employment | jobs in denver |
| Job-Employment | jobs in orlando |
| Job-Employment | jobs in san diego |
| Job-Employment | jobs in tucson |
| Job-Employment | jobs in utah |
| Job-Employment | jobs near |
| Job-Employment | jobs nyc |
| Job-Employment | jobs orlando |
| Job-Employment | jobs part time |
| Job-Employment | jobs? |
| Job-Employment | jobseeker |
| Job-Employment | kansas unemployment rate |
| Job-Employment | kentucky unemployment rate |
| Job-Employment | ks unemployment |
| Job-Employment | ky unemployment |
| Job-Employment | lay off |
| Job-Employment | laying off |
| Job-Employment | layoff |
| Job-Employment | layoff news |
| Job-Employment | layoff tracker |
| Job-Employment | layoffs |
| Job-Employment | length of unemployment |
| Job-Employment | length of unemployment benefits |
| Job-Employment | ma unemployment |
| Job-Employment | management jobs |
| Job-Employment | maryland unemployment rate |
| Job-Employment | mass unemployment |
| Job-Employment | maximum unemployment |
| Job-Employment | maximum unemployment benefits |
| Job-Employment | medical assistant |
| Job-Employment | michigan uia |
| Job-Employment | missouri unemployment |
| Job-Employment | missouri unemployment rate |
| Job-Employment | mn unemployment |
| Job-Employment | my unemployment benefits |
| Job-Employment | national unemployment rate |
| Job-Employment | nc unemployment rate |
| Job-Employment | nevada unemployment |
| Job-Employment | nevada unemployment rate |
| Job-Employment | nj unemployment extension |
| Job-Employment | nj unemployment rate |
| Job-Employment | no job |
| Job-Employment | non-profit jobs |
| Job-Employment | north carolina unemployment rate |
| Job-Employment | nv unemployment |
| Job-Employment | nys unemployment |
| Job-Employment | obama unemployment |
| Job-Employment | oregon unemployment |
| Job-Employment | pa unemployment |
| Job-Employment | paralegal jobs |
| Job-Employment | pharmacy technician |
| Job-Employment | recent layoffs |
| Job-Employment | recruiters |
| Job-Employment | resume for |
| Job-Employment | retail jobs |
| Job-Employment | sales associate |
| Job-Employment | sc jobs |
| Job-Employment | sc unemployment rate |
| Job-Employment | secretary jobs |
| Job-Employment | security jobs |
| Job-Employment | security officer |
| Job-Employment | service jobs |
| Job-Employment | simply hired |
| Job-Employment | state unemployment |
| Job-Employment | store manager |
| Job-Employment | tech jobs |
| Job-Employment | temporary employment |
| Job-Employment | tennessee unemployment |
| Job-Employment | texas employment |
| Job-Employment | texas unemployment |
| Job-Employment | the unemployed |
| Job-Employment | therapy jobs |
| Job-Employment | tier 2 unemployment |
| Job-Employment | training centers |
| Job-Employment | tutoring jobs |
| Job-Employment | tx jobs |
| Job-Employment | ui benefits |
| Job-Employment | unemp |
| Job-Employment | unemploy |
| Job-Employment | unemployed |
| Job-Employment | unemployed benefits |
| Job-Employment | unemployed workers |
| Job-Employment | unemployment |
| Job-Employment | unemployment |
| Job-Employment | unemployment benefit |
| Job-Employment | unemployment benefits |
| Job-Employment | unemployment benefits by state |
| Job-Employment | unemployment benefits in california |
| Job-Employment | unemployment benefits in illinois |
| Job-Employment | unemployment blog |
| Job-Employment | unemployment by state |
| Job-Employment | unemployment chart |
| Job-Employment | unemployment check |
| Job-Employment | unemployment direct deposit |
| Job-Employment | unemployment eligibility |
| Job-Employment | unemployment extended |
| Job-Employment | unemployment extended benefits |
| Job-Employment | unemployment in |
| Job-Employment | unemployment in california |
| Job-Employment | unemployment length |
| Job-Employment | unemployment obama |
| Job-Employment | unemployment online |
| Job-Employment | unemployment rate |
| Job-Employment | unemployment rate by county |
| Job-Employment | unemployment rate history |
| Job-Employment | unemployment rate in california |
| Job-Employment | unemployment rate in new york |
| Job-Employment | unemployment rates by state |
| Job-Employment | us unemployment |
| Job-Employment | us unemployment benefits |
| Job-Employment | us unemployment rate |
| Job-Employment | us unemployment rate history |
| Job-Employment | va unemployment |
| Job-Employment | waitress jobs |
| Job-Employment | what is minimum wage |
| Job-Employment | who is eligible for unemployment |
| Job-Employment | who is eligible for unemployment benefits |
| Job-Employment | who qualifies for unemployment |
| Job-Employment | wi unemployment |
| Job-Employment | wisconsin unemployment |
| Job-Employment | worksource |
| Money-Spending | "irs |
| Money-Spending | 10 year treasury note |
| Money-Spending | 1040 |
| Money-Spending | 1040 a |
| Money-Spending | 1040 online |
| Money-Spending | 1040 schedule |
| Money-Spending | 1040 schedule a |
| Money-Spending | 1040 tax return |
| Money-Spending | 1040 tax table |
| Money-Spending | 1040a |
| Money-Spending | 1040ex |
| Money-Spending | 1040ez |
| Money-Spending | 1040ez form |
| Money-Spending | 1040ez online |
| Money-Spending | 1099-r |
| Money-Spending | 2441 |
| Money-Spending | 30 year mortgage rate |
| Money-Spending | 5329 |
| Money-Spending | aaa fuel gauge |
| Money-Spending | aaa gas |
| Money-Spending | aaa gas prices |
| Money-Spending | apartment guide |
| Money-Spending | area gas prices |
| Money-Spending | arizona department of revenue |
| Money-Spending | arizona dept of revenue |
| Money-Spending | average family income |
| Money-Spending | average household income |
| Money-Spending | average income |
| Money-Spending | bank prime |
| Money-Spending | bank prime rate |
| Money-Spending | bank rates |
| Money-Spending | barrel of crude |
| Money-Spending | barrel of gas |
| Money-Spending | barrel of oil |
| Money-Spending | barrel of oil price |
| Money-Spending | barrel oil price |
| Money-Spending | barrel oil prices |
| Money-Spending | barrel price |
| Money-Spending | best gas price |
| Money-Spending | best gas prices |
| Money-Spending | bloomberg energy |
| Money-Spending | bloomberg oil |
| Money-Spending | bloomberg oil price |
| Money-Spending | bloomberg oil prices |
| Money-Spending | budget battle |
| Money-Spending | budget breakdown |
| Money-Spending | budget congress |
| Money-Spending | budget cut |
| Money-Spending | budget deal |
| Money-Spending | budget debate |
| Money-Spending | budget deficit |
| Money-Spending | budget federal |
| Money-Spending | budget us |
| Money-Spending | canada gas |
| Money-Spending | canada gas prices |
| Money-Spending | cheap gas |
| Money-Spending | cheap gas price |
| Money-Spending | cheap gas prices |
| Money-Spending | cheap gas stations |
| Money-Spending | cheap gas.com |
| Money-Spending | cheaper gas |
| Money-Spending | cheapest gas |
| Money-Spending | cheapest gas price |
| Money-Spending | cheapest gas prices |
| Money-Spending | cheapest gas stations |
| Money-Spending | cheapgas.com |
| Money-Spending | chicago apartment |
| Money-Spending | city income tax |
| Money-Spending | city tax |
| Money-Spending | coldwell banker |
| Money-Spending | colorado department of revenue |
| Money-Spending | colorado gas |
| Money-Spending | colorado tax |
| Money-Spending | congress budget |
| Money-Spending | connecticut department of revenue |
| Money-Spending | cost of barrel of oil |
| Money-Spending | crude oil |
| Money-Spending | crude oil chart |
| Money-Spending | crude oil per barrel |
| Money-Spending | crude oil price |
| Money-Spending | crude oil prices |
| Money-Spending | crude oil prices today |
| Money-Spending | crude oil stock |
| Money-Spending | crude oil stock symbol |
| Money-Spending | crude oil symbol |
| Money-Spending | crude oil today |
| Money-Spending | crude price |
| Money-Spending | crude prices |
| Money-Spending | ct drs |
| Money-Spending | current budget |
| Money-Spending | current crude |
| Money-Spending | current crude oil |
| Money-Spending | current crude oil price |
| Money-Spending | current crude oil prices |
| Money-Spending | current gas price |
| Money-Spending | current inflation |
| Money-Spending | current interest |
| Money-Spending | current interest rate |
| Money-Spending | current interest rates |
| Money-Spending | current mortgage interest |
| Money-Spending | current mortgage interest rates |
| Money-Spending | current mortgage rate |
| Money-Spending | current oil |
| Money-Spending | current oil price |
| Money-Spending | current oil prices |
| Money-Spending | current price of crude |
| Money-Spending | current price of crude oil |
| Money-Spending | current price of oil |
| Money-Spending | current prime |
| Money-Spending | current prime interest rate |
| Money-Spending | current prime rate |
| Money-Spending | current rates |
| Money-Spending | daily oil prices |
| Money-Spending | deficit |
| Money-Spending | deficit spending |
| Money-Spending | deficits |
| Money-Spending | delaware tax |
| Money-Spending | department of revenue |
| Money-Spending | department of revenue services |
| Money-Spending | department of tax |
| Money-Spending | department of taxation |
| Money-Spending | department of treasury |
| Money-Spending | dept revenue |
| Money-Spending | dept. of revenue |
| Money-Spending | diesel fuel |
| Money-Spending | diesel prices |
| Money-Spending | division of taxation |
| Money-Spending | e file |
| Money-Spending | easement |
| Money-Spending | education spending |
| Money-Spending | efile taxes |
| Money-Spending | epa budget |
| Money-Spending | equity loan rates |
| Money-Spending | equity rates |
| Money-Spending | era realty |
| Money-Spending | estate services |
| Money-Spending | european gas prices |
| Money-Spending | ez 1040 |
| Money-Spending | fair housing |
| Money-Spending | family income |
| Money-Spending | family income |
| Money-Spending | fed budget |
| Money-Spending | fed fund |
| Money-Spending | fed fund rate |
| Money-Spending | fed funds |
| Money-Spending | fed funds rate |
| Money-Spending | fed funds rates |
| Money-Spending | fed interest |
| Money-Spending | fed interest rate |
| Money-Spending | fed interest rates |
| Money-Spending | fed prime rate |
| Money-Spending | fed rate |
| Money-Spending | fed rates |
| Money-Spending | fed tax |
| Money-Spending | fed tax forms |
| Money-Spending | fed taxes |
| Money-Spending | federal 1040 |
| Money-Spending | federal adjusted gross income |
| Money-Spending | federal budget |
| Money-Spending | federal budget breakdown |
| Money-Spending | federal budget cuts |
| Money-Spending | federal budget deficit |
| Money-Spending | federal deficit |
| Money-Spending | federal form |
| Money-Spending | federal fund |
| Money-Spending | federal fund rate |
| Money-Spending | federal funds |
| Money-Spending | federal funds rate |
| Money-Spending | federal government budget |
| Money-Spending | federal government shutdown |
| Money-Spending | federal interest |
| Money-Spending | federal interest rate |
| Money-Spending | federal interest rates |
| Money-Spending | federal prime rate |
| Money-Spending | federal rate |
| Money-Spending | federal reserve interest |
| Money-Spending | federal reserve interest rate |
| Money-Spending | federal reserve interest rates |
| Money-Spending | federal reserve prime rate |
| Money-Spending | federal reserve rate |
| Money-Spending | federal reserve rates |
| Money-Spending | federal spending |
| Money-Spending | federal tax |
| Money-Spending | federal tax filing |
| Money-Spending | federal tax form 1040 |
| Money-Spending | federal taxes |
| Money-Spending | file federal taxes |
| Money-Spending | file income tax |
| Money-Spending | file state tax |
| Money-Spending | file tax return |
| Money-Spending | file taxes online |
| Money-Spending | filing federal taxes |
| Money-Spending | filing state taxes |
| Money-Spending | filing tax return |
| Money-Spending | filing taxes |
| Money-Spending | find cheap gas |
| Money-Spending | find cheapest gas |
| Money-Spending | find gas prices |
| Money-Spending | find lowest gas prices |
| Money-Spending | first realty |
| Money-Spending | fixed interest |
| Money-Spending | fixed interest rate |
| Money-Spending | fixed interest rates |
| Money-Spending | fixed rate |
| Money-Spending | fixed rates |
| Money-Spending | form 1040 |
| Money-Spending | form 1040 instructions |
| Money-Spending | form 1040a |
| Money-Spending | form 1040ez |
| Money-Spending | form 2106 |
| Money-Spending | form 2441 |
| Money-Spending | form 5329 |
| Money-Spending | form schedule a |
| Money-Spending | forms 1040 |
| Money-Spending | free state tax |
| Money-Spending | fuel gauge report |
| Money-Spending | fuel price |
| Money-Spending | fuel prices |
| Money-Spending | fuel surcharge |
| Money-Spending | fuel surcharges |
| Money-Spending | furnished apartment |
| Money-Spending | future gas prices |
| Money-Spending | future of gas prices |
| Money-Spending | gas cheap |
| Money-Spending | gas price |
| Money-Spending | gas price finder |
| Money-Spending | gas price in usa |
| Money-Spending | gas price locator |
| Money-Spending | gas price per barrel |
| Money-Spending | gas price prediction |
| Money-Spending | gas price projection |
| Money-Spending | gas price projections |
| Money-Spending | gas price tracker |
| Money-Spending | gas prices |
| Money-Spending | gas prices aaa |
| Money-Spending | gas prices high |
| Money-Spending | gas prices in |
| Money-Spending | gas prices in america |
| Money-Spending | gas prices in canada |
| Money-Spending | gas prices in europe |
| Money-Spending | gas prices in hawaii |
| Money-Spending | gas prices in the united states |
| Money-Spending | gas prices in the us |
| Money-Spending | gas prices in united states |
| Money-Spending | gas prices in usa |
| Money-Spending | gas prices news |
| Money-Spending | gas prices per barrel |
| Money-Spending | gas prices us |
| Money-Spending | gas prices usa |
| Money-Spending | gas prices? |
| Money-Spending | gas prices.com |
| Money-Spending | gas pump prices |
| Money-Spending | gas station prices |
| Money-Spending | gasoline price |
| Money-Spending | gov tax forms |
| Money-Spending | government budget |
| Money-Spending | government budget cuts |
| Money-Spending | government cuts |
| Money-Spending | government expenditures |
| Money-Spending | government freeze |
| Money-Spending | government revenue |
| Money-Spending | government spending |
| Money-Spending | hawaii gas prices |
| Money-Spending | heloc |
| Money-Spending | high gas |
| Money-Spending | high gas mileage |
| Money-Spending | high gas price |
| Money-Spending | high gas prices |
| Money-Spending | highest gas price |
| Money-Spending | highest gas prices |
| Money-Spending | history of gas prices |
| Money-Spending | home equity loan rates |
| Money-Spending | home equity rates |
| Money-Spending | home interest |
| Money-Spending | home interest rate |
| Money-Spending | home interest rates |
| Money-Spending | home loan |
| Money-Spending | home loan interest |
| Money-Spending | home loan rate |
| Money-Spending | home mortgage interest |
| Money-Spending | home mortgage interest rates |
| Money-Spending | home rates |
| Money-Spending | home realty |
| Money-Spending | homes |
| Money-Spending | homes sold |
| Money-Spending | house budget |
| Money-Spending | household income |
| Money-Spending | housing |
| Money-Spending | illinois dept of revenue |
| Money-Spending | income distribution |
| Money-Spending | income levels |
| Money-Spending | income tax |
| Money-Spending | income tax returns |
| Money-Spending | income us |
| Money-Spending | index rate |
| Money-Spending | indiana department of revenue |
| Money-Spending | inflation |
| Money-Spending | interest rate |
| Money-Spending | interest rate mortgage |
| Money-Spending | interest rate news |
| Money-Spending | interest rates |
| Money-Spending | interest rates home |
| Money-Spending | internal revenue |
| Money-Spending | internal revenue service |
| Money-Spending | iowa state tax |
| Money-Spending | irs 1040 |
| Money-Spending | irs 1040a |
| Money-Spending | irs 1040ez |
| Money-Spending | irs audit |
| Money-Spending | irs ez |
| Money-Spending | irs ez form |
| Money-Spending | irs form |
| Money-Spending | irs form 1040 |
| Money-Spending | irs form 1040ez |
| Money-Spending | irs forms |
| Money-Spending | irs instructions |
| Money-Spending | irs schedule a |
| Money-Spending | irs tax returns |
| Money-Spending | kentucky tax |
| Money-Spending | ky state tax |
| Money-Spending | ky tax |
| Money-Spending | latest oil prices |
| Money-Spending | lending rate |
| Money-Spending | lending rates |
| Money-Spending | light sweet crude |
| Money-Spending | light sweet crude oil |
| Money-Spending | light sweet crude price |
| Money-Spending | live oil prices |
| Money-Spending | loan rate |
| Money-Spending | local gas |
| Money-Spending | local gas price |
| Money-Spending | local gas prices |
| Money-Spending | local tax |
| Money-Spending | lofts |
| Money-Spending | low gas |
| Money-Spending | low gas price |
| Money-Spending | low gas prices |
| Money-Spending | lower gas prices |
| Money-Spending | lowest gas |
| Money-Spending | lowest gas price |
| Money-Spending | lowest gas prices |
| Money-Spending | maryland comptroller |
| Money-Spending | maryland income tax |
| Money-Spending | maryland state income tax |
| Money-Spending | maryland state tax |
| Money-Spending | maryland tax |
| Money-Spending | median household income |
| Money-Spending | median income |
| Money-Spending | mexico gas prices |
| Money-Spending | michigan department of treasury |
| Money-Spending | military budget |
| Money-Spending | minimum income |
| Money-Spending | minnesota department of revenue |
| Money-Spending | minnesota tax |
| Money-Spending | minnesota taxes |
| Money-Spending | mississippi state tax |
| Money-Spending | missouri department of revenue |
| Money-Spending | mn state tax |
| Money-Spending | mn tax |
| Money-Spending | mortgage |
| Money-Spending | mortgage interest |
| Money-Spending | mortgage interest rate |
| Money-Spending | mortgage interest rates |
| Money-Spending | mortgage loan rates |
| Money-Spending | mortgage rate |
| Money-Spending | most expensive gas |
| Money-Spending | motorcycle gas mileage |
| Money-Spending | national budget |
| Money-Spending | national deficit |
| Money-Spending | national gas |
| Money-Spending | nebraska tax |
| Money-Spending | new york department of taxation |
| Money-Spending | new york state department of taxation |
| Money-Spending | new york state taxation |
| Money-Spending | new york tax |
| Money-Spending | nih budget |
| Money-Spending | north carolina department of revenue |
| Money-Spending | north carolina dept of revenue |
| Money-Spending | north carolina taxes |
| Money-Spending | nymex crude |
| Money-Spending | nymex crude future |
| Money-Spending | nymex crude oil |
| Money-Spending | nymex oil |
| Money-Spending | ohio state taxes |
| Money-Spending | ohio taxation |
| Money-Spending | oil barrel |
| Money-Spending | oil barrel price |
| Money-Spending | oil barrel prices |
| Money-Spending | oil chart |
| Money-Spending | oil close |
| Money-Spending | oil closing price |
| Money-Spending | oil crude |
| Money-Spending | oil per barrel |
| Money-Spending | oil price |
| Money-Spending | oil price barrel |
| Money-Spending | oil price chart |
| Money-Spending | oil price graph |
| Money-Spending | oil price history |
| Money-Spending | oil price news |
| Money-Spending | oil price per barrel |
| Money-Spending | oil prices |
| Money-Spending | oil quote |
| Money-Spending | oil spot |
| Money-Spending | oil spot price |
| Money-Spending | oil ticker |
| Money-Spending | oil today |
| Money-Spending | oklahoma state tax |
| Money-Spending | oklahoma tax |
| Money-Spending | oklahoma tax commission |
| Money-Spending | planned parenthood funding |
| Money-Spending | price barrel of oil |
| Money-Spending | price barrel oil |
| Money-Spending | price for barrel of oil |
| Money-Spending | price gas |
| Money-Spending | price of a barrel of oil |
| Money-Spending | price of barrel of oil |
| Money-Spending | price of crude |
| Money-Spending | price of crude oil |
| Money-Spending | price of gasoline |
| Money-Spending | price of oil |
| Money-Spending | price of oil per barrel |
| Money-Spending | price of oil today |
| Money-Spending | price oil |
| Money-Spending | price oil barrel |
| Money-Spending | price per barrel |
| Money-Spending | price per barrel of oil |
| Money-Spending | price per gallon |
| Money-Spending | prime interest |
| Money-Spending | prime interest rate |
| Money-Spending | prime interest rates |
| Money-Spending | prime lending |
| Money-Spending | prime lending rate |
| Money-Spending | prime rate |
| Money-Spending | prime rate history |
| Money-Spending | prime rate? |
| Money-Spending | prime rates |
| Money-Spending | projected gas prices |
| Money-Spending | property |
| Money-Spending | proposed budget cuts |
| Money-Spending | rate mortgage |
| Money-Spending | rate of inflation |
| Money-Spending | real estate services |
| Money-Spending | real property |
| Money-Spending | real time oil prices |
| Money-Spending | realtors in |
| Money-Spending | realty |
| Money-Spending | realty inc |
| Money-Spending | realty world |
| Money-Spending | reserve rate |
| Money-Spending | return filing |
| Money-Spending | revenue |
| Money-Spending | revenue department |
| Money-Spending | revenue dept |
| Money-Spending | revenue service |
| Money-Spending | revenue services |
| Money-Spending | rising gas prices |
| Money-Spending | schedule eic |
| Money-Spending | school district tax |
| Money-Spending | south carolina department of revenue |
| Money-Spending | south carolina taxes |
| Money-Spending | spot oil |
| Money-Spending | standard of living |
| Money-Spending | state department of taxation |
| Money-Spending | state efile |
| Money-Spending | state filing |
| Money-Spending | state form |
| Money-Spending | state forms |
| Money-Spending | state housing |
| Money-Spending | state income |
| Money-Spending | state income tax |
| Money-Spending | state income tax return |
| Money-Spending | state income taxes |
| Money-Spending | state irs |
| Money-Spending | state returns |
| Money-Spending | state revenue |
| Money-Spending | state tax |
| Money-Spending | state tax department |
| Money-Spending | state tax filing |
| Money-Spending | state tax returns |
| Money-Spending | state taxation |
| Money-Spending | statutory employee |
| Money-Spending | stock market oil |
| Money-Spending | sweet crude |
| Money-Spending | sweet crude oil |
| Money-Spending | sweet crude price |
| Money-Spending | symbol for crude oil |
| Money-Spending | t bill |
| Money-Spending | tariffs |
| Money-Spending | tax |
| Money-Spending | tax audit |
| Money-Spending | tax codes |
| Money-Spending | tax commission |
| Money-Spending | tax department |
| Money-Spending | tax dept |
| Money-Spending | tax e file |
| Money-Spending | tax efile |
| Money-Spending | tax filing |
| Money-Spending | tax form 1040 |
| Money-Spending | tax form 1040ez |
| Money-Spending | tax forms 1040 |
| Money-Spending | tax information |
| Money-Spending | tax irs |
| Money-Spending | tax return |
| Money-Spending | tax return filing |
| Money-Spending | tax returns |
| Money-Spending | tax site |
| Money-Spending | tax state |
| Money-Spending | tax website |
| Money-Spending | taxable interest |
| Money-Spending | taxation |
| Money-Spending | taxation department |
| Money-Spending | taxes |
| Money-Spending | taxes free |
| Money-Spending | taxes online |
| Money-Spending | taxes state |
| Money-Spending | the federal budget |
| Money-Spending | the price of oil |
| Money-Spending | the prime rate |
| Money-Spending | the us budget |
| Money-Spending | today oil price |
| Money-Spending | townhome |
| Money-Spending | townhouses |
| Money-Spending | treasury note |
| Money-Spending | u.s. budget |
| Money-Spending | u.s. deficit |
| Money-Spending | united states budget |
| Money-Spending | united states deficit |
| Money-Spending | us budget |
| Money-Spending | us budget deficit |
| Money-Spending | us federal budget |
| Money-Spending | us gas |
| Money-Spending | us gas price |
| Money-Spending | us gas prices |
| Money-Spending | us government budget |
| Money-Spending | us government spending |
| Money-Spending | us inflation |
| Money-Spending | us interest rate |
| Money-Spending | us prime |
| Money-Spending | us prime rate |
| Money-Spending | virginia department of revenue |
| Money-Spending | virginia department of taxation |
| Money-Spending | virginia income tax |
| Money-Spending | virginia state tax |
| Money-Spending | virginia tax |
| Money-Spending | virginia taxes |
| Money-Spending | w-2g |
| Money-Spending | wage tax |
| Money-Spending | wall street journal prime |
| Money-Spending | wall street journal prime rate |
| Money-Spending | wall street prime |
| Money-Spending | wall street prime rate |
| Money-Spending | west virginia tax |
| Money-Spending | what is prime rate |
| Money-Spending | what is the prime rate |
| Money-Spending | which tax form |
| Money-Spending | who has to file |
| Money-Spending | who must file |
| Money-Spending | wisconsin department of revenue |
| Money-Spending | wisconsin income tax |
| Money-Spending | wisconsin state tax |
| Money-Spending | wisconsin tax |
| Money-Spending | wisconsin taxes |
| Money-Spending | wsj prime |
| Money-Spending | wsj prime rate |
