# Supplementary material for: Interplay between Public Attention and Public Emotion toward Multiple Social Issues on Twitter
Source: PLoS One. 2017 Jan 12;12(1):e0167896. doi: 10.1371/journal.pone.0167896 (PMC5231282; doi:10.1371/journal.pone.0167896)
Supplement: S2 Table — (DOCX) [file pone.0167896.s002.docx]

S2 Table. Bivariate Granger-Causality Test Results

| Dependent Variables | Independent Variables | | | | | | | | | | | | | | |
| --- | --- | --- | --- | --- | --- | --- | --- | --- | --- | --- | --- | --- | --- | --- | --- |
|  | 1 | 2 | 3 | 4 | 5 | 6 | 7 | 8 | 9 | 10 | 11 | 12 | 13 | 14 | 15 |
| 1. Attention_Economy |  | 0.590 | 0.783 | 0.444 | 0.600 | 0.377 | 0.513 | 0.561 | 0.085 | 0.334 | 0.166 | 0.245 | 0.119 | 0.449 | 0.059 |
| 2. Attention_Politics | 0.174 |  | 0.603 | 0.847 | 0.436 | 0.154 | 0.378 | 0.808 | 0.621 | 0.071 | 0.562 | 0.425 | 0.051 | 0.521 | 0.479 |
| 3. Attention_Health | 0.131 | 0.450 |  | 0.549 | 0.824 | 0.008 | 0.517 | 0.52 | 0.983 | 0.149 | 0.810 | 0.580 | 0.938 | 0.219 | 0.594 |
| 4. Attention_Employment | 0.000 | 0.944 | 0.175 |  | 0.101 | 0.942 | 0.198 | 0.256 | 0.133 | 0.581 | 0.619 | 0.950 | 0.007 | 0.915 | 0.757 |
| 5. Attention_Spending | 0.696 | 0.864 | 0.135 | 0.175 |  | 0.879 | 0.041 | 0.552 | 0.329 | 0.012 | 0.255 | 0.323 | 0.024 | 0.765 | 0.014 |
| 6. Arousal_Economy | 0.318 | 0.613 | 0.882 | 0.795 | 0.507 |  | 0.014 | 0.116 | 0.551 | 0.760 | 0.090 | 0.430 | 0.956 | 0.667 | 0.124 |
| 7. Arousal_Politics | 0.513 | 0.604 | 0.857 | 0.928 | 0.103 | 0.406 |  | 0.147 | 0.057 | 0.590 | 0.961 | 0.028 | 0.703 | 0.697 | 0.038 |
| 8. Arousal_Health | 0.604 | 0.534 | 0.562 | 0.194 | 0.615 | 0.630 | 0.198 |  | 0.033 | 0.929 | 0.316 | 0.307 | 0.233 | 0.240 | 0.133 |
| 9. Arousal_Employment | 0.825 | 0.088 | 0.122 | 0.156 | 0.716 | 0.583 | 0.004 | 0.050 |  | 0.421 | 0.950 | 0.100 | 0.243 | 0.860 | 0.009 |
| 10. Arousal_Spending | 0.718 | 0.698 | 0.808 | 0.417 | 0.938 | 0.828 | 0.788 | 0.440 | 0.968 |  | 0.729 | 0.586 | 0.585 | 0.164 | 0.628 |
| 11. Valence_Economy | 0.534 | 0.108 | 0.114 | 0.474 | 0.282 | 0.211 | 0.878 | 0.621 | 0.924 | 0.268 |  | 0.854 | 0.916 | 0.739 | 0.219 |
| 12. Valence_Politics | 0.318 | 0.642 | 0.304 | 0.046 | 0.940 | 0.318 | 0.083 | 0.531 | 0.834 | 0.815 | 0.577 |  | 0.104 | 0.819 | 0.010 |
| 13. Valence_Health | 0.610 | 0.842 | 0.201 | 0.337 | 0.488 | 0.006 | 0.494 | 0.155 | 0.109 | 0.404 | 0.438 | 0.065 |  | 0.433 | 0.753 |
| 14. Valence_Employment | 0.153 | 0.191 | 0.293 | 0.067 | 0.578 | 0.170 | 0.042 | 0.226 | 0.044 | 0.421 | 0.404 | 0.935 | 0.280 |  | 0.037 |
| 15. Valence_Spending | 0.338 | 0.300 | 0.659 | 0.153 | 0.674 | 0.563 | 0.438 | 0.589 | 0.184 | 0.066 | 0.319 | 0.831 | 0.081 | 0.040 |  |

Note: The numbers reported are the probability values of the bivariate Granger-causality tests with time lag equal to 1.

If the probability value is smaller than 0.10, we can conclude that there is a Granger-causality relationship between two variables.
